# Directed evolution expands CRISPR–Cas12a genome-editing capacity

**DOI:** 10.1093/nar/gkaf649

**Published:** 2025-07-16

**Authors:** Enbo Ma, Kai Chen, Honglue Shi, Kevin M Wasko, Isabel Esain-Garcia, Marena I Trinidad, Kaihong Zhou, Jinjuan Ye, Jennifer A Doudna

**Affiliations:** Innovative Genomics Institute, University of California, Berkeley, Berkeley, CA 94720, United States; Department of Molecular and Cell Biology, University of California, Berkeley, Berkeley, CA 94720, United States; Innovative Genomics Institute, University of California, Berkeley, Berkeley, CA 94720, United States; Department of Molecular and Cell Biology, University of California, Berkeley, Berkeley, CA 94720, United States; Innovative Genomics Institute, University of California, Berkeley, Berkeley, CA 94720, United States; Howard Hughes Medical Institute, University of California, Berkeley, Berkeley, CA 94720, United States; Innovative Genomics Institute, University of California, Berkeley, Berkeley, CA 94720, United States; Department of Molecular and Cell Biology, University of California, Berkeley, Berkeley, CA 94720, United States; Innovative Genomics Institute, University of California, Berkeley, Berkeley, CA 94720, United States; California Institute for Quantitative Biosciences (QB3), University of California, Berkeley, Berkeley, CA 94720, United States; Innovative Genomics Institute, University of California, Berkeley, Berkeley, CA 94720, United States; California Institute for Quantitative Biosciences (QB3), University of California, Berkeley, Berkeley, CA 94720, United States; Howard Hughes Medical Institute, University of California, Berkeley, Berkeley, CA 94720, United States; Howard Hughes Medical Institute, University of California, Berkeley, Berkeley, CA 94720, United States; Innovative Genomics Institute, University of California, Berkeley, Berkeley, CA 94720, United States; Department of Molecular and Cell Biology, University of California, Berkeley, Berkeley, CA 94720, United States; Howard Hughes Medical Institute, University of California, Berkeley, Berkeley, CA 94720, United States; California Institute for Quantitative Biosciences (QB3), University of California, Berkeley, Berkeley, CA 94720, United States; Molecular Biophysics and Integrated Bioimaging Division, Lawrence Berkeley National Laboratory, Berkeley, CA 94720, United States; Li Ka Shing Center for Genomic Engineering, University of California, Berkeley, Berkeley, CA 94720, United States; Department of Chemistry, University of California, Berkeley, Berkeley, CA 94720, United States; Gladstone Institute of Data Science and Biotechnology, San Francisco, CA 94158, United States; Gladstone-UCSF Institute of Genomic Immunology, San Francisco, CA 94158, United States

## Abstract

CRISPR–Cas12a enzymes are versatile RNA-guided genome-editing tools with applications encompassing viral diagnosis, agriculture, and human therapeutics. However, their dependence on a 5′-TTTV-3′ protospacer adjacent motif (PAM) next to DNA target sequences restricts Cas12a’s gene targeting capability to only ∼1% of a typical genome. To mitigate this constraint, we used a bacterial-based directed evolution assay combined with rational engineering to identify variants of *Lachnospiraceae bacterium* Cas12a with expanded PAM recognition. The resulting Cas12a variants use a range of noncanonical PAMs while retaining recognition of the canonical 5′-TTTV-3′ PAM. In particular, biochemical and cell-based assays show that the variant Flex-Cas12a utilizes 5′-NYHV-3′ PAMs that expand DNA recognition sites to ∼25% of the human genome. With enhanced targeting versatility, Flex-Cas12a unlocks access to previously inaccessible genomic loci, providing new opportunities for both therapeutic and agricultural genome engineering.

## Graphical Abstract

**Graphical Abstract:**
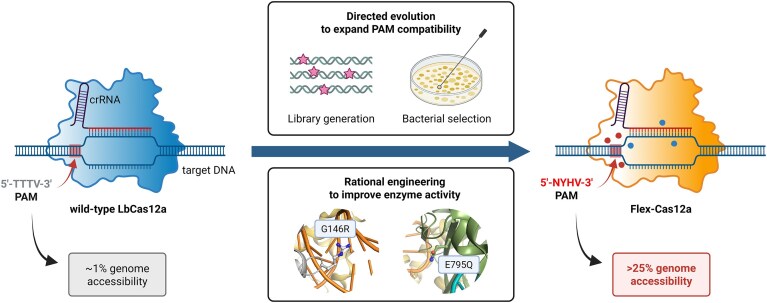

## Introduction

CRISPR–Cas12a is an RNA-guided endonuclease used for genome editing in plants, animals, and human cells [1–4]. It employs self-processed CRISPR RNAs (crRNAs) to locate target DNA sequences in the genome [1, 5]. Target recognition by Cas12a relies on a 5′-TTTV-3′ (V = A, C, or G) protospacer adjacent motif (PAM) as a key signal that enables crRNA-mediated DNA binding and formation of an RNA–DNA R-loop spanning a 20-bp target sequence. R-loop formation triggers Cas12a-mediated target DNA cleavage [1, 6, 7], inducing site-specific sequence changes during the DNA repair process.

Although Cas12a is less widely used than Cas9 [8, 9], its unique properties—including its low-temperature-tolerant ability to generate staggered double-strand breaks and autonomously process crRNA arrays—make it particularly attractive for applications in plants [10, 11] and for multiplexed genome targeting [1, 5, 12]. Furthermore, engineered Cas12a enzymes with improved nuclease activity have expanded their utility across diverse cell types and organisms [13–16]. However, Cas12a’s stringent requirement for a four-nucleotide 5′-TTTV-3′ PAM limits targetable sites to ∼1% of a typical genome [17]. In comparison, the canonical Cas9 from *Streptococcus pyogenes* (SpyCas9), which requires only a 5′-NGG-3′ PAM, can access >6% of the genome [17]. The stringent PAM dependency stems from CRISPR’s natural immune function in bacteria, where PAM recognition helps distinguish foreign DNA from the host genome, ensuring selective cleavage of invasive genetic fragments. Efforts to relax PAM requirements through structure-guided engineering of Cas9 and Cas12a enzymes have generated variants with expanded targeting capabilities [17, 18, 19, 20]. However, these modifications often reduce cleavage kinetics or increase off-target activity, likely due to slower and more promiscuous target search mechanisms [21–23].

To address these limitations, we explored whether a comprehensive mutational approach could lead to Cas12a variants with relaxed PAM requirements and retained genome-editing efficiency. It has been shown that *Lachnospiraceae bacterium* Cas12a (LbCas12a) exhibits high nuclease activity and is broadly used in both mammalian and plant systems with higher target specificity and relatively low sensitivity to temperature compared to other Cas12a orthologs [24–28]. Moreover, our group has previously achieved successful enhancement of LbCas12a activity through directed evolution, demonstrating its amenability to protein engineering [15]. Therefore, we chose LbCas12a as an optimal starting point for PAM reprogramming in this study. By combining directed evolution and rational engineering, we generated LbCas12a variants with both expanded PAM tolerance and robust nuclease activity. Among these, we identified Flex-Cas12a, a standout variant carrying six mutations (G146R, R182V, D535G, S551F, D665N, and E795Q). Flex-Cas12a not only retains efficient cleavage activity at canonical 5′-TTTV-3′ sites but also recognizes a range of PAM sequences (5′-NYHV-3′), thereby expanding potential genome accessibility from ∼1% to over 25%. These improvements provide new tools for genome engineering across diverse biological systems, advancing both fundamental and translational applications.

## Materials and methods

### Library generation of LbCas12a variants with random mutations in the PAM-interacting and wedge domains

**Figure 1.**
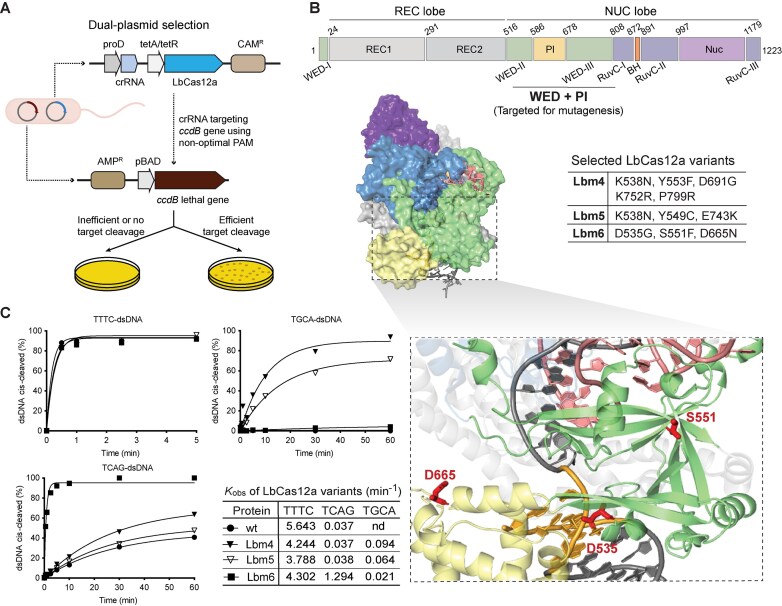
Generation of PAM-relaxed Cas12a enzymatic variants. (**A**) Schematic presentation of the dual-plasmid selection system used for directed evolution. Each of the four crRNAs (crRNA1–4) was designed for targeting sequences flanked by randomly designed noncanonical PAMs of 5′-AGCT-3′, 5′-AGTC-3′, 5′-TGCA-3′, or 5′-TCAG-3′. All DNA target and crRNA sequences are listed in Supplementary Table S1. (**B**) Schematic presentation of the LbCas12a functional domains. The highlighted middle region (WED + PI, top panel) is the target region for mutagenesis. Overall structure (PDB: 5XUS) of LbCas12a–dsDNA–crRNA ternary complex [40] is shown on the left side of the middle panel, while mutations in three selected variants are listed in the table on the right side of the middle panel. The bottom panel displays the structural positions of the mutations of D535G, S551F, and D665N in Lbm6, with the three mutated residues highlighted. The PAM sequence is highlighted in the structure. (**C**) *In vitro* kinetic studies of *cis-*cleavage activity on different PAM DNAs. Cleavage efficiencies of wild-type (wt) LbCas12a and three selected variants (Lbm4, Lbm5, and Lbm6) are shown. Each data point represents the average of two independent experiments. DNA substrates used in these assays are DNA T0 (listed in Supplementary Table S1) with different PAM sequences. 5′-TTTC-3′ is a canonical PAM, while others represent noncanonical PAMs. Each PAM sequence is listed on the top of each panel. The observed rate constants (*k*_obs_) from these *cis*-cleavage assays are also listed. Here, nd stands for not detectable.

For directed evolution (Fig. 1A), we chose the region containing both PI (PAM-interacting) and WED (wedge) domains from LbCas12a to generate libraries using random mutagenesis (Fig. 1B), since these two domains interact with the PAM and its surrounding nucleotides [6, 7, 29, 30].

To generate a library with random mutations in this target region, we performed error-prone polymerase chain reaction (PCR) to amplify the DNA fragment encoding PI and WED-II/III domains (Fig. 1B) as described elsewhere [15, 31]. To limit the error rate to 6–9-nucleotide mutations per kilobase, 2.4 μl of 10 mM MnCl_2_ was added to a 100 μl PCR reaction (total volume) that contained 10 μl of 10× ThermoPol reaction buffer, 2 μl of 10 mM primers, 30 ng of template plasmid, and 1 μl of ThermoTaq DNA Polymerase (M0267S, NEB). The resulting error-prone PCR products were then used to replace the corresponding wild-type fragment in an LbCas12a bacterial expression plasmid via Gibson assembly.

### Selection of PAM-relaxed LbCas12a variants using directed evolution

To isolate LbCas12a variants with relaxed PAM requirements, we carried out directed evolution using a dual-bacterial selection system described previously (Fig. 1A) [15, 32–35]. The generation of LbCas12a mutant libraries bearing random mutations in specific domains follows the procedures described in our previous work [15]. Briefly, we constructed four independent chloramphenicol-resistant (CAM^+^) bacterial expression libraries, each harboring the mutagenized PI and WED-II/III domains along with a specific crRNA. Each library contains ∼10^5^ Cas12a variants, generated through error-prone PCR. In the bacterial expression vector, LbCas12a is under TetR promoter whereas crRNA is under a strong consecutive promoter proD. Each crRNA was designed to direct cleavage at a target sequence adjacent to a noncanonical PAM (crRNA1: AGCT; crRNA2: AGTC; crRNA3: TGCA; crRNA4: TCAG). These PAMs and target sites were randomly chosen within the *ccdB* lethal gene located in the selection plasmid that contains an ampicillin-resistant (Amp^+^) gene (Fig. 1A; sequences listed in Supplementary Fig. S1A and Supplementary Table S1). The sequences of primers used to generate Cas12a libraries with random mutations in the PI and WED-II/III domains and corresponding loci in the *ccdB* lethal gene are listed in Supplementary Table S1. For each library, 2 ng of plasmid DNA was electroporated into 50 μl of *E. coli* strain *BW25141*(*DE3*) competent cells containing an ampicillin-resistant (Amp^+^) plasmid encoding an arabinose-inducible *ccdB* lethal gene. The expression of the *ccdB* lethal gene is tightly controlled by the promoter pBAD, which can be induced with arabinose (2 mM) for positive selection (Fig. 1A). After recovery of electroporated bacteria in 2 ml of SOB for 40 min at 37°C, 5 μl of the bacterial culture was plated onto a CAM-containing Petri agar dish (as a control) and the remaining culture was plated on another Petri agar dish containing both arabinose and CAM. Positive colonies that grew on plates containing arabinose and CAM were collected and replated. Plasmids from individual replated colonies were then isolated and sequenced. In this study, two rounds of selection were performed to enrich for LbCas12a variants with relaxed PAM specificity. Corresponding plasmids used in this study will be deposited to Addgene.

### Protein expression and purification

The expression and purification of LbCas12a proteins tagged with 2× NLS on the N-terminus and 4× NLS on the C-terminus were carried out using the CL7/Im7 expression and purification protocol (Trialtus Bioscience) with some modifications. Briefly, the coding sequences for each LbCas12 variant were cloned into a dual-tagged (His/CL7) expression vector and transformed into *E. coli* BL21(DE3) cells (NEB). Cells were cultured in 2× YT medium (Thermo Fisher Scientific) supplemented with ampicillin (100 μg/ml) at 37°C until its optical density (OD_600_) reached 0.6–0.8 (using an overnight starter culture at a 1:40 ratio). The cultures were then cooled on ice for 30–60 min before induction with 0.5 mM isopropyl β-d-1-thiogalactoside (IPTG). Following IPTG induction, cells were grown overnight at 16°C. To purify the Cas12a proteins, the cultured cells were harvested and resuspended in lysis buffer [50 mM Tris–HCl (pH 7.5), 20 mM imidazole, 1 M NaCl, 10% (v/v) glycerol and supplemented with 1 mM tris(2-carboxyethyl)phosphine (TCEP) and a cOmplete™ Protease Inhibitor Cocktail Tablet (Millipore Sigma) for every 50 ml]. Lysis was conducted by sonication. The lysed cultures were clarified by centrifugation for 60 min at 18 000 × *g*. The clarified supernatant was then applied to Ni-NTA resin (Qiagen), which was pre-equilibrated with wash buffer [50 mM Tris–HCl (pH 7.5), 20 mM imidazole, 0.5 mM TCEP, 1 M NaCl]. The mixture was then incubated for 30–60 min at 4°C to allow binding of the His-tagged target protein to Ni-NTA resin, then washed three times with the same buffer, and finally eluted with a buffer containing 50 mM Tris–HCl (pH 7.5), 300 mM imidazole, 1 M NaCl, 10% glycerol, and 1 mM TCEP. The Ni-NTA resin-purified proteins were applied two to three times to Im7 beads. The beads were then washed with 5–10 column volumes of a wash buffer containing 20 mM HEPES (pH 7.5), 1 M NaCl, 10% glycerol, and 1 mM TCEP before addition of Pierce human rhinovirus (HRV) 3C protease (Thermo Fisher Scientific) to release the LbCas12a protein. The solution containing the target protein was collected and then concentrated with a 30 000 MWCO concentrator (Millipore Sigma). The concentrated protein was further purified by application to a Superdex 200 Increase 10/300 GL (Cytiva) column using gel filtration buffer [20 mM HEPES (pH 7.5), 150 mM KCl, 10% glycerol, and 1 mM TCEP]. Peak fractions containing Cas12a proteins were collected, concentrated, and quantified using a NanoDrop 8000 Spectrophotometer (Thermo Fisher Scientific), and stored at −80°C after flash-freezing with liquid nitrogen.

### Nucleic acid preparation

All DNA and RNA oligos used for *in vitro* experiments in this study were purchased from Integrated DNA Technologies, Inc. (IDT) and HPLC- or PAGE-purified. For genome editing experiments, crRNAs contained chemical modifications at their 3′- or 5′-ends to improve stability and editing efficiency in cells (detailed in IDT guidance).

DNA substrate (T0, shown as nontarget strand) used for *in vitro* cleavage assays is 5′-GACGACAAAAC**NNNN**GATCGTTACGCTAACTATGAGGGCTGTCTGTGGAATGCTA-3′ (here, NNNN is a PAM sequence and listed in each corresponding figure). Guide RNA of crRNA0 used for *in vitro* cleavage assays is 5′-AAUUUCUACUCUUGUAGAUGAUCGUUACGCUAACUAUGAGGGC-3′.

Four DNA targets used in the directed evolution selection are listed below (bold letters indicate noncanonical PAM sequences in front of the protospacers that are underlined): T1: **AGCT**TTCATCCCCGATATGCACCACCGG; T2: **AGTC**TCCCGTGAACTTTACCCGGTGGTG; T3: **TGCA**TATCGGGGATGAAAGCTGGCGCAT; and T4: **TCAG**ATAAAGTCTCCCGTGAACTTTACC.

Their corresponding guide RNAs (crRNAs) used for the selection are crRNA1, crRNA2, crRNA3, and crRNA4, respectively.

The sequences of all DNAs and RNAs used for *in vitro* assays in this study are also listed in Supplementary Table S1.

### DNA cleavage assays

Typical *cis*-cleavage assays (otherwise will be stated) were carried out in a buffer containing 60 nM protein, 72 nM of crRNA, and 10 nM of 5′-FAM-labeled nontarget strand of a target dsDNA in cleavage buffer [20 mM HEPES (pH 7.5), 150 mM KCl, 10 mM MgCl_2_, 1% glycerol, and 1 mM TCEP]. Specifically, the protein and a guide crRNA were first incubated for 15 min at room temperature to form RNPs and then incubated at 37°C for a specified duration of time after the addition of labeled target DNA. For *trans*-cleavage assays, the reaction conditions were the same as *cis*-cleavage reactions except that 45 nM unlabeled target dsDNA instead of labeled target dsDNA was used. After incubation for 30 min at 37°C, a labeled random ssDNA (no homology with the target DNAs or crRNAs) was added to the reactions. The reactions were continually incubated for certain durations of time. The reactions of both *cis*- and *trans*-cleavage were quenched with 1× DNA loading buffer (45% formamide, 15 mM EDTA, trace amount of xylene cyanol and bromophenol blue). After denaturation at 95°C for 3 min, the cleavage products were separated using a 15% urea–PAGE gel and quantified using a Typhoon phosphorimager (Amersham, GE Healthcare). Cleavage kinetics were determined by fitting time-course data to the equation


\begin{eqnarray*}
{Y} = {{{Y}}_{{\mathrm{max}}}} \times \left( {1 - {{{\mathrm{e}}}^{ - kt}}} \right),
\end{eqnarray*}

where ${{{Y}}_{{\mathrm{max}}}}$ is the pre-exponential factor, *k* is the rate constant (min^−1^), and *t* is the reaction time (min).

### PAM depletion assays

A single DNA oligo containing a randomized 6N PAM region (at each position, N = A, C, G, or T) in front of the Cas12a target sequence was synthesized by IDT. The single DNA oligos were annealed and primer-extended into dsDNA oligos using PCR polymerase. To generate a random PAM plasmid library, the dsDNA oligos were subsequently Gibson-cloned into a pUC19 plasmid backbone.

PAM depletion assays were performed under conditions similar to the standard DNA cleavage assays described above. Briefly, 60 nM wt LbCas12a or its variants were pre-incubated with 72 nM crRNA complementary to the corresponding protospacer sequence, which is downstream of the 6N PAM region in the plasmid library (protospacer and guide RNA sequences are listed in Supplementary Table S1). The Cas12a–crRNA complex was then added to the plasmid library (13 nM) and incubated at 37°C for 10 min. The reaction was quenched by addition of one volume of 2× quench buffer (95% formamide and 30 mM EDTA) and heat treatment for 10 min at 95°C. The plasmid DNAs were purified using magnetic beads. After cleanup, the PAM region in the uncleaved plasmid DNAs was amplified by PCR in the presence of the primers containing partial Illumina adapter sequences (primer sequences are listed in Supplementary Table S1) that allow for subsequent indexing in the second-round PCR. The first-round PCR reactions were treated with the addition of 0.5 μl of DpnI for 30 min at 37°C before purification using magnetic beads. The purified PCR products were subjected to the indexed PCR to make indexed libraries. The indexed libraries were pooled equimolarly, quantified by Qubit, and sequenced on an Illumina NextSeq platform (P1 flow cell) with 2 × 150 bp paired-end reads (IGI NGS sequencing core, UC Berkeley). The sequencing run typically achieved sufficient coverage (> 500 000 reads per library) to provide a comprehensive view of PAM preferences across the 6N library.

Sequencing data were processed according to the high-throughput PAM determination assay (HT-PAMDA) pipeline [36] using a customized Python script provided at https://github.com/kleinstiverlab/HT-PAMDA. In brief, raw FASTQ reads were aligned to a reference sequence to extract the 6-nt PAM regions. For each PAM, its frequency in the Cas12a RNP-treated sample was compared to that in a control sample (plasmid without treatment with any RNP) to calculate a relative depletion rate after a single 10-min reaction. These depletion rate constants (expressed on a log scale) serve as a quantitative measure of cleavage activity at each PAM site. The resulting heatmaps display the log rate constants using a color gradient, where darker hues indicate higher cleavage activity (or greater depletion) and lighter hues denote lower activity.

### Cell culture

HEK293T cells (UC Berkeley Cell Culture Facility) were cultured using Dulbecco’s modified of Eagle’s medium (DMEM) with l-glutamine, 4.5 g/l glucose and sodium pyruvate (Corning) plus 10% FBS, and penicillin and streptomycin (Gibco). Neural progenitor cells (NPCs) were isolated from the brains of Ai9-tdTomato homozygous mice at embryonic day 13.5. Cells were cultured as neurospheres in NPC medium [DMEM/F12 with glutamine, sodium pyruvate, 10 mM HEPES, non-essential amino acids, 1× penicillin and streptomycin, 1× 2-mercaptoethanol, B-27 without vitamin A, N2 supplement, and growth factors, bFGF and EGF (both 20 ng/ml at final concentration)]. NPCs were passaged using the MACS Neural Dissociation Kit (Papain, CAT# 130-092-628) following the manufacturer’s protocol. bFGF and EGF were refreshed every 3 days, and cells were passaged every 6 days. The NPCs were authenticated by immunocytochemistry marker staining for Nestin and GFAP.

### Genome editing

To evaluate the genome-editing activity of LbCas12a variants, we electroporated the corresponding ribonucleoproteins (RNPs) into regular HEK293T cells, EGFP-expressing HEK293T cells (HEK293T-EGFP), or tdTomato-transgene-containing NPCs. For each transfection, 100 pmol of LbCas12a protein was pre-incubated with 120 pmol crRNA at room temperature for 15–25 min to form RNPs, after which 80 pmol of a ssDNA electroporation enhancer (purchased from IDT) was added.

For HEK293T or HEK293T-EGFP cells, RNP nucleofection was carried out with Lonza (Allendale, NJ) SF cell kits in an Amaxa 96-well Shuttle system (program code CM-130). Each nucleofection reaction consisted of ∼2.0 × 10^5^ cells and 100 pmol RNP in a total volume of 20 μl of supplemented SF buffer following the manufacturer’s instructions. After nucleofection, 120 μl of growth medium was added to each nucleofection cuvette, and 5000–10 000 cells were subsequently transferred to individual wells of a 96-well tissue culture plate preloaded with 100–120 μl of full medium. After incubation at 37°C for 3 days, the cell culture medium was refreshed. After an additional 3 days of culture, flow cytometry was used to determine editing efficiency, as indicated by loss of either EGFP or B2M.

Briefly, HEK293T cells were detached by addition of 30 μl 0.05% trypsin–EDTA per well and incubated for ∼3 min before the addition of 120 μl complete media to each well. One hundred twenty microliters of the treated cells were transferred to a 96-well U-bottom plate, to which 80 μl of PBS was added in each well and centrifuged at 1600 rpm for 5 min. After the media were decanted, the cells were washed with 150 μl PBS/BSA (1% BSA). The washed cells were resuspended in 50 μl of staining master mix containing anti-B2M-APC (Biolegend, Luxembourg; 1:200 dilution with PBS supplemented with 1% BSA), or resuspended in PBS to be analyzed directly, when assessing EGFP knockout. The cells were stained for 30 min in the dark before the addition of 100 μl PBS/BSA for washing. Following an additional wash with 100 μl PBS/BSA, the cells were resuspended in 150 μl PBS and analyzed by flow cytometry using an Attune Flow Cytometer (Thermo Fisher Scientific).

Nucleofection of NPCs with RNPs was performed using Lonza (Allendale, NJ) P3 cell kits in an Amaxa 96-well Shuttle system with program code EH-100. Each nucleofection reaction consisted of ∼2.5 × 10^5^ cells and 100 pmol RNP [preassembled in a phosphate buffer (25 mM NaPi, 300 mM NaCl, 200 mM trehalose, pH 7.5)] with a total volume of 20 μl in the supplemented P3 buffer following the manufacturer’s instructions. After nucleofection, 80 μl of growth medium was added to the nucleofection cuvette, and 5 μl of NPC culture was then transferred to 96-well tissue culture plates pre-coated (using laminin, fibronectin, and poly-dl-ornithine) with a total culture volume of 100 μl/well. Following a 3-day incubation at 37°C, the medium was refreshed, and the cells were harvested after an additional 3 days for FACS of tdTomato expression .

To further evaluate the genome-editing activity of LbCas12a variants, we also performed plasmid transfection using 4.0 × 10^4^ cells in 96-well plates. Specifically, 100 ng of LbCas12a-expressing plasmid (under a CMV promoter) was co-transfected with either 50 or 100 ng of a crRNA-encoding plasmid (under a human U6 promoter) using Lipofectamine 3000 (Thermo Fisher). To analyze EGFP knockdown efficiency, transfected EGFP-HEK cells were harvested 6 days post-transfection for FACS analysis. For NGS analysis of on-target and off-target editing, HEK293T cells were collected 4 days post-transfection, and genomic DNA was isolated using 100 μl of Quick Extraction solution, as described in the protocols below (see the “NGS sequencing” section). In this study, we selected two previously published endogenous sites of DNMT1 (sites 3 and 7) [17] for on- and off-target analyses (Supplementary Table S2).

### Base editing

We constructed both cytosine base editor (CBE) and adenine base editor (ABE) versions of LbCas12a by modifying two existing plasmid backbones. The CBE backbone [13] is derived from pCAG-NLS(SV40)x2-rAPOBEC1-gs-XTEN-gs-hdLbCas12a(D832A)-gs-UGI-NLS(SV40) (LbBE1.4, Addgene plasmid #114086, RTW1293). The ABE backbone [37] is made from pCMV-dCas12a-ABE8e (Addgene plasmid #193645). LbCas12a base editors (BEs) were constructed by cloning human codon-optimized LbCas12a variants (synthesized as gBlocks, Twist Bioscience) into the respective vectors for mammalian expression.

Each BE plasmid was co-transfected with a corresponding crRNA-encoding plasmid driven by the human U6 promoter. We designed four crRNA constructs for cytosine base editing and four crRNA constructs for adenine base editing; their target sequences and PAMs are listed in Supplementary Table S3. For each transfection, 100 ng of the BE plasmid and 40 ng of the crRNA plasmid were introduced into 2.0 × 10⁴ HEK293T cells plated in 96-well plates. Transfections were carried out using FuGENE transfection reagent according to the manufacturer’s recommended protocol (Promega). On day 4 post-transfection, genomic DNA was isolated according to the protocols described below.

### NGS sequencing

To produce amplicons used in NGS, all genomic DNAs were extracted following the protocol supplied in the Quick Extraction solution (Epicentre, Madison, WI). Briefly, after the culture medium was removed, 100 μl of Quick Extraction solution was added to each well to lyse the cells (65°C for 20 min and then 95°C for 20 min). The concentration of genomic DNA was determined by NanoDrop and was stored at −20°C until use.

DNA sequences of potential off-targets were identified as described elsewhere [30]. All off-target predictions were generated using Cas-OFFinder [38] with parameters allowing up to five mismatches and one bulge, designed to capture a broad spectrum of potential off-target sites. Briefly, we used Cas-OFFinder (v3.0.0b3) and the human reference genome GRCh38.p14 to identify potential off-target sequences for two target sites with a canonical PAM of TTTA or a noncanonical PAM of TCAG, respectively. The parameters used in this study to select potential off-target sites are the sequences with ≤1 bulge, ≤5 mismatches, and specific PAMs.

To confirm genome editing based on B2M knockout, to investigate off-target editing by the LbCas12a variants in HEK293T cells, and to analyze the efficiency of base editing, target or off-target amplicons were PCR-amplified in the presence of corresponding primers. Target loci were first amplified by a first PCR step using locus-specific primers (primer sequences are listed in Supplementary Table S4), and indexed libraries were generated through a second PCR step to append Illumina adapter sequences and unique sample barcodes. The PCR products were purified with magnetic beads (Berkeley Sequencing Core Facility) and then were pooled at equimolar concentrations, quantified, and sequenced on an Illumina NextSeq platform (P1 flow cell) with 2 × 150 bp paired-end reads at the IGI NGS sequence core, resulting in sufficient coverage of the amplicon (>10 000 reads per library).

Data were analyzed with Geneious Prime (v2024.07) (https://www.geneious.com/) and CRISPResso2 (v2.2.6) [39] for indel quantification, with a 50% minimum alignment score and two biological replicates for indel analysis and three biological replicates for BE analysis. Paired-end Illumina sequencing reads were initially processed in Geneious Prime (https://www.geneious.com/prime) using the BBDuk module to remove low-quality bases (minimum score of 20) and discard reads shorter than 20 nucleotides. The filtered reads were then merged with BBmerge, also within Geneious Prime, to produce contiguous sequences where possible. For samples that included only forward (R1) reads, no merging step was performed. After preprocessing, the resulting reads were analyzed in CRISPResso2 (https://github.com/pinellolab/CRISPResso2) to quantify both indel formation and base editing frequencies. Two commands were used, one focusing on indel rates and another on base editing outcomes:

CRISPResso --fastq_r1 MERGED_READS --amplicon_seq AMPLICON_SEQUENCE --guide_seq GUIDE_SEQUENCE -n nhej -wc -3 -w 5 --plot_window_size 20 -o OUTPUT_FILE

CRISPResso --fastq_r1 MERGED_READS --amplicon_seq AMPLICON_SEQUENCE --guide_seq GUIDE_SEQUENCE -n nhej -wc -12 -w 6 --plot_window_size 20 --base_editor_output -o OUTPUT_FILE

In these commands, *--fastq_r1* points to the processed FASTQ file (merged or unmerged), while *--amplicon_seq* indicates the entire amplified region being examined. The *--guide_seq* parameter specifies the guide RNA spacer. The flags *-wc* and *-w* set the center and size of the quantification window relative to the 3′ end of the guide RNA, and *--plot_window_size* affects the visualization range for indels. When base editing was examined, *--base_editor_output* was included, allowing CRISPResso2 to report C-to-T or A-to-G substitutions over the defined window. This workflow provided a clear assessment of both indel formation and base editing efficiency for the targeted loci.

## Results

### Directed evolution of LbCas12a variants with relaxed PAM requirements

The requirement for T-rich PAMs (5′-TTTV-3′, where V is A, C, or G) in LbCas12a limits its applications in genome editing [1, 8, 17]. To overcome this limitation, we employed a bacterial selection-based directed evolution system [15, 33–35] to generate LbCas12a variants capable of recognizing a wide spectrum of PAM sequences (Fig. 1A). Guided by previous findings that the PI and WED domains in both Cas12a and Cas9 are critical for PAM binding and DNA interaction [6, 7, 30], we targeted the PI, WED-II, and WED-III regions of LbCas12a for random mutagenesis via error-prone PCR (Fig. 1B, top panel).

We arbitrarily designed four guide crRNAs (crRNA1–4) to target DNA sequences flanked by noncanonical PAMs of 5′-AGCT-3′, 5′-AGTC-3′, 5′-TGCA-3′, and 5′-TCAG-3′, respectively (Supplementary Fig. S1A). These DNA target sequences are located within the coding sequence of the lethal protein ccdB in the selection plasmid [15] (Fig. 1A). Following two sequential rounds of selection, seven PAM-relaxed LbCas12a variants (designated Lbm1 to Lbm7) emerged from the libraries corresponding to crRNA2, crRNA3, and crRNA4 (Fig. 1B, middle panel, right, and Supplementary Fig. S1B). Specifically, four LbCas12a variants (Lbm1–4) were selected from crRNA2, one (Lbm5) from crRNA3, and two (Lbm6–7) from crRNA4 (Supplementary Fig. S1B). Interestingly, no variant was obtained with crRNA1, likely because the corresponding 5′-AGCT-3′ PAM resembles part of the crRNA scaffold (5′-AGAU-3′), which could lead to self-targeting of the crRNA1 locus. Most of the mutations in the selected LbCas12a variants occurred in the WED-II and -III domains (Supplementary Fig. S1B). The finding that mutations in the WED domains are involved in the alteration of LbCas12a PAM-binding capabilities (Fig. 1B, middle panel, right, and Supplementary Fig. S1B) underscores the potential of WED domains to affect target recognition [6, 7, 30, 41]. Structural mapping of these mutations onto the known LbCas12a ternary structure showed that while a few mutations were positioned near PAM-recognition sites, others were found in regions previously implicated in crRNA binding or general DNA interactions (Fig. 1B, bottom panel, and Supplementary Fig. S1C). This diverse mutational landscape contrasts with structure-guided rational engineering targeting residues directly involved in PAM binding [13, 17–20, 30]. Instead, this random mutagenesis approach aimed to uncover beneficial mutations across multiple protein regions, thereby expanding the potential for engineering Cas12a variants with relaxed PAM requirements.

The seven LbCas12a variant proteins were expressed and purified (Supplementary Fig. S1D) to biochemically confirm their PAM recognition preferences. Based on biochemical cleavage activities using both short synthetic dsDNA (Supplementary Fig. S1E) and plasmid substrates (Supplementary Fig. S1F), we chose three variants (Lbm4-6) for detailed kinetic analysis with dsDNA substrates bearing various PAMs. All three variants exhibited cleavage rates comparable to those measured for wt LbCas12a when tested with a canonical 5′-TTTV-3′ PAM-containing substrate (*k*_obs_ = 3.8–5.6 min^−1^) and could recognize noncanonical PAMs as well (Fig. 1C). Additional cleavage assays revealed that Lbm6 had the most relaxed PAM specificity (Supplementary Fig. S1G). Given its expanded target range and robust biochemical activity, we focused on Lbm6 in subsequent studies.

### Construction of a composite genome editor with a relaxed PAM requirement

**Figure 2.**
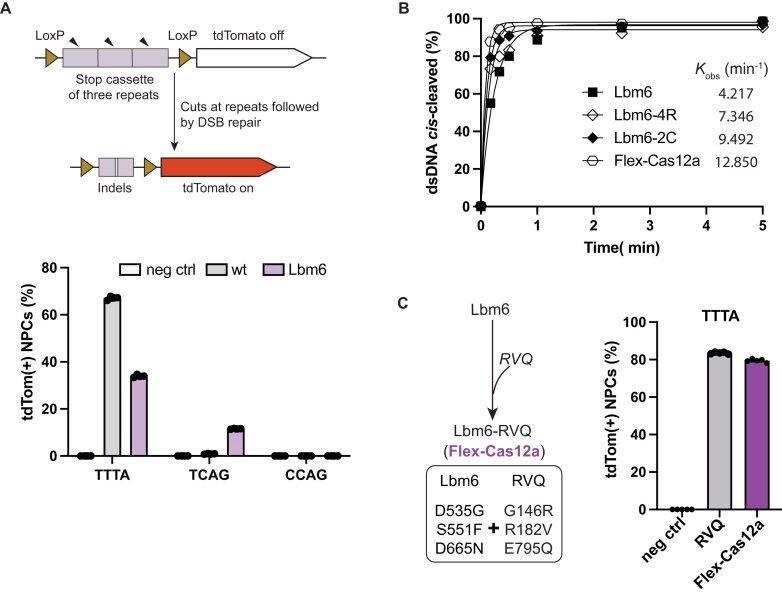
Nuclease activities of Lbm6 and its derivatives. (**A**) Genome editing in Ai9 mouse-derived NPCs. The upper panel provides a schematic illustration of the desired genome editing to turn on the tdTomato transgene in NPCs. The lower panel presents genome-editing efficiencies at three target sites in NPCs. PAMs are shown on the *X*-axis. TTTA is a canonical PAM, while others represent noncanonical PAMs. Each bar represents the average of four independent experiments. (**B**) Improvement of Lbm6 enzymatic cleavage activity. *cis*-Cleavage activity of Lbm6 is improved by introduction of the mutations from the activity-enhanced variant of Cas12a-4R, -2C, or -RVQ. Introduction of RVQ mutations (G146R, R182V, and E795Q) into Lbm6 makes it most active, as shown by its *k*_obs_. From now on, the combined version of Lbm6 and RVQ mutations will be referred to as Flex-Cas12a. Each data point represents the average of two independent experiments. DNA target used in this assay is DNA T0 with a canonical PAM of 5′-TTTC-3′. (**C**) Genome-editing efficiency of Flex-Cas12a. Flex-Cas12a exhibits significantly enhanced genome-editing activity, which is comparable to LbCas12a-RVQ, at a locus with a canonical PAM of 5′-TTTA-3′ in tdTomato NPCs. Negative control (neg ctrl) conditions in panels (A) and (C) mean cells that were not treated by RNP. Each bar represents the average of four independent technical replicates.

To assess the genome-editing activity of variant Lbm6, we first employed tdTomato reporter NPCs derived from Ai9 mice [42, 43] and delivered Cas12a RNPs via nucleofection (Fig. 2A, upper panel). In this system, genome editing at the transgene locus to remove the stop cassette upstream of the tdTomato gene activates tdTomato expression. Six days post-nucleofection of RNPs, Lbm6, but not wt LbCas12a, successfully edited loci flanked by noncanonical PAMs (Fig. 2A, lower panel). However, at a target site bearing a canonical 5′-TTTA-3′ PAM, Lbm6 generated half the level of editing (34%) as that observed with wt LbCas12a (67%) (Fig. 2A, lower panel). The lower genome-editing rate shown in Lbm6 suggested that mutations in this variant might compromise its activity.

Previous studies showed that modifications in either the NUC or REC lobe of Cas12a can significantly enhance its nuclease activity [14–16]. Based on these findings, we tested whether Lbm6’s editing activity could be improved by incorporating known activity-enhancing mutations. Three sets of mutations from Cas12a-RVQ [16], hyperCas12a [14], or iCas12a [15] were introduced into Lbm6, resulting in the new variants Lbm6-RVQ, Lbm6-4R, and Lbm6-2C, respectively. Biochemical DNA cleavage assays demonstrated that Lbm6-RVQ displayed the highest activity among the three Lbm6 derivatives (Fig. 2B and Supplementary Fig. S2A and B). We tested the genome-editing activity of this variant in tdTomato NPCs at a locus with a 5′-TTTA-3′ PAM. At this locus, Lbm6-RVQ achieved an 80% editing rate, substantially higher than its parental variant Lbm6 (34%) and wt LbCas12a (67%), and comparable to LbCas12a-RVQ (84%) (Fig. 2C). Henceforth, we refer to Lbm6-RVQ as Flex-Cas12a in this study.

**Figure 3.**
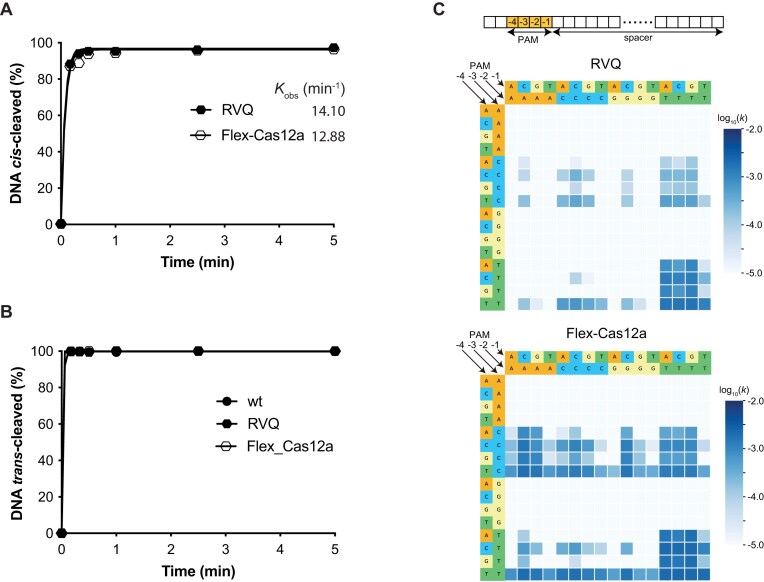
*In vitro* kinetic studies and PAM depletion assays. (**A**) *In vitro* kinetic analysis of *cis*-cleavage activity. The observed *k*_obs_ indicate that Flex-Cas12a exhibits cleavage activity comparable to LbCas12a-RVQ (RVQ). Each data point represents the average of two independent experiments. The target DNA used in this assay is DNA T0 (listed in Supplementary Table S1) with a canonical PAM of 5′-TTTC-3′. (**B**) *In vitro* kinetic analysis of *trans*-cleavage activity. Due to the rapid cleavage rates, the *k*_obs_ of *trans*-cleavage could not be accurately determined from these assays. Each data point represents the average of two independent experiments. Target DNA and guide RNA used in this assay are listed in Supplementary Table S1. (**C**) Expanded PAM recognition by Flex-Cas12a. Heatmaps of NGS results from PAM depletion assays illustrate PAM preferences of LbCas12a-RVQ (RVQ) and Flex-Cas12a. Top panel presents a schematic illustration of nucleotide positions in the PAM motif. Middle panel shows the heatmap for LbCas12a-RVQ, while bottom panel depicts the heatmap for Flex-Cas12a. The heatmap represents the log_10_(*k*) of the *in vitro* cleavage rate constant (s^−1^).

Given that Cas12a enzymes typically possess both *cis*-cleavage activity and target-activated *trans*-DNA cutting activity [44, 45], we also tested both *cis*- and *trans*-cleavage efficiencies of Flex-Cas12a in comparison with LbCas12a-RVQ. We found that Flex-Cas12a maintained similar *cis*- and *trans*-cleavage activities to LbCas12a-RVQ when tested with canonical 5′-TTTV-3′ PAM targets (Fig. 3A and B, and Supplementary Fig. S3A and B).

To systematically analyze the PAM recognition profile of Flex-Cas12a, we performed PAM-depletion assays. While, as previously reported [16], LbCas12a-RVQ primarily recognizes canonical 5′-TTTV-3′ PAMs (Fig. 3C, middle panel), Flex-Cas12a utilizes a wide range of PAMs, specifically those with sequence 5′-NYHV-3′ (Y is T or C, H is A, C, or T, and N is any base) (Fig. 3C, bottom panel). This expanded recognition enables Flex-Cas12a to potentially target >25% of a human genome, extending the ∼6% accessibility achieved at maximum by previous LbCas12a variants [17, 18, 20].

### Specificity of genome editing by Flex-Cas12a in different cell types

**Figure 4.**
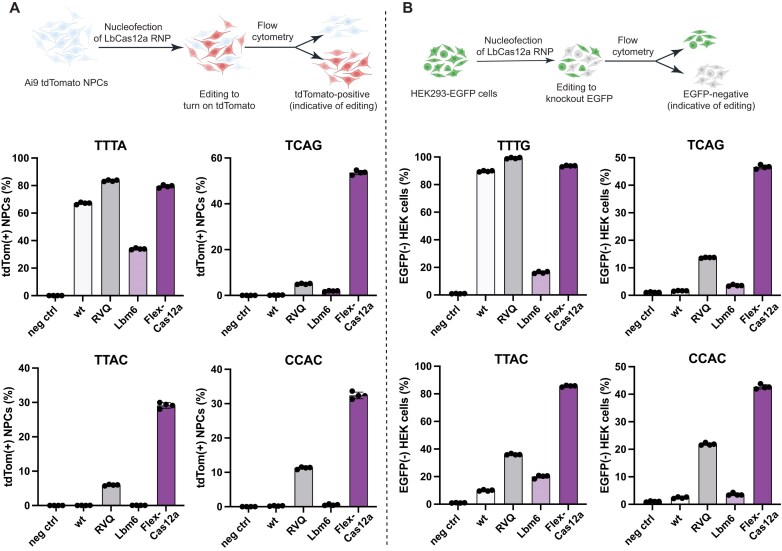
Genome editing in Ai9 tdTomato NPCs and HEK293T-EGFP cells. (**A**) Genome editing in tdTomato NPCs. The upper panel provides a schematic illustration of the desired genome editing to turn on the tdTomato transgene in NPCs. The lower panels show the genome-editing efficiencies at four loci by wt, LbCas12a-RVQ (RVQ), Lbm6, and Flex-Cas12a. PAM sequence of each target is shown on top of each panel. (**B**) Genome editing in HEK293T-EGFP cells. The upper panel provides a schematic illustration of the desired genome editing to turn off the EGFP transgene in HEK293T cells. The lower panels show the genome-editing efficiencies of four proteins at four loci. TTTA and TTTG are canonical PAMs, while others represent noncanonical PAMs. The PAM sequence of each target is shown on top of each panel. The genome-editing results from both cell types demonstrate that Flex-Cas12a is significantly more active than its parent variant (Lbm6) and can efficiently recognize a broad range of PAM sequences for genome editing. Data are presented as mean ± SD from four independent technical replicates.

To evaluate the utility of Flex-Cas12a at target sites with different PAMs in different mammalian cell types, we conducted genome-editing assays in HEK293T-EGFP cells and tdTomato-expressing mouse NPCs [15, 30, 46]. For these assays, we designed crRNAs targeting 10 different reporter genomic loci bearing various noncanonical PAMs in HEK293-EGFP cells and 10 such targets in tdTomato NPCs. Six days post-nucleofection of cells with corresponding RNPs, flow cytometry analysis showed that Flex-Cas12a edited these targets at levels ranging from 60% to 80% in both cell types, whereas LbCas12a-RVQ exhibited minimal activity at the loci with noncanonical PAMs (Fig. 4A and B, and Supplementary Fig. S4A and B). To further validate these findings, we performed plasmid transfections at two concentrations (150 and 200 ng; see the “Materials and methods” section for details). The editing results from plasmid delivery showed trends consistent with those observed using RNPs (Supplementary Fig. S4C and D). These results indicate that Flex-Cas12a can effectively recognize 5′-NYHV-3′ PAMs, significantly expanding its genome-editing capability beyond that displayed by wt and engineered LbCas12a enzymes [17, 18, 20, 47, 48].

**Figure 5.**
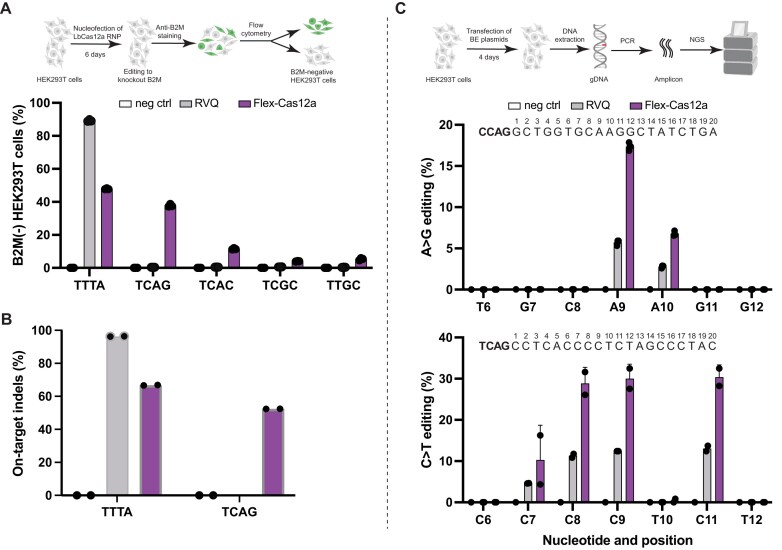
Genome editing at endogenous loci. (**A**) Genome editing at endogenous loci in HEK293T cells. The upper panel illustrates the detection of genome editing in HEK293T cells using an anti-B2M antibody. The lower panel shows editing efficiencies at five endogenous loci in the B2M gene. Flex-Cas12a can edit five endogenous loci with different PAMs, whereas LbCas12a-RVQ (RVQ) is restricted to the site with a canonical PAM. TTTA is a canonical PAM, while others represent noncanonical PAMs. The PAM sequence of each target is listed on the *X*-axis. (**B**) Genome-editing analysis by NGS. Editing efficiencies at two selected targets with a canonical PAM of 5′-TTTA-3′ and a noncanonical PAM of 5′-TCAG-3′ are shown. The data presented here are generated from three independent technical replicates. Indels here represent insertions and deletions. (**C**) Base editing analysis by NGS analysis. Top panel is a schematic illustration of the analysis of base editing. Middle panel presents representative data from ABE. Bottom panel shows representative data from CBE. Target DNA sequences are listed on the top of each panel, corresponding PAMs are highlighted in bold, and each position of nucleotide after PAM is also numbered. All target sequences, their corresponding genes, and primers used for base editing analysis are listed in Supplementary Table S3. All the data are presented as mean ± SD from three independent technical replicates.

Next, we assessed the ability of Flex-Cas12a RNPs to edit endogenous genomic loci by designing crRNAs to target five sites in the human β2-microglobulin gene, encoding a broadly expressed surface protein with clinical relevance for cell therapies [49]. Knockout was monitored using a B2M-specific monoclonal antibody that indicates successful gene disruption [50]. Six days post-nucleofection with Flex-Cas12a RNPs, flow cytometry analysis showed editing efficiencies ranging from 5% to 40% at genomic sites containing either a canonical or noncanonical PAM. The variable editing outcomes of Flex-Cas12a can be due to locus-dependent factors, such as chromatin accessibility, local sequence context, or crRNA stability. In contrast, similar experiments conducted using LbCas12a-RVQ RNPs showed editing only at sites containing canonical 5′-TTTV-3′ PAMs (Fig. 5A). NGS confirmed that Flex-Cas12a achieved ∼50% editing using a noncanonical TCAG PAM sequence, whereas LbCas12a-RVQ showed minimally detectable activity at this site (Fig. 5B), while no notable difference in the indel profiles was observed between Flex-Cas12a and RVQ (Supplementary Fig. S4E). These results were further supported by results from additional endogenous sites (Supplementary Fig. S5A). Notably, no off-target activity was detected following RNP delivery at sites predicted by Cas-OFFinder [38] (Supplementary Fig. S5B and C); this is presumably due to the transient presence of RNP in the cellular environment [51–53]. To further validate these findings, we performed plasmid transfections targeting two previously characterized DNMT1 loci (sites 3 and 7) with known off-target sites [17] (Supplementary Table S2). NGS analysis revealed significantly reduced off-target editing levels by Flex-Cas12a compared to LbCas12a-RVQ (*P* < .01, Supplementary Fig. S5D). Collectively, these results demonstrate that Flex-Cas12a retains robust on-target activity while exhibiting reduced off-target effects relative to LbCas12a-RVQ.

Finally, we tested whether Flex-Cas12a could be employed for base editing at target sites with noncanonical PAMs. We constructed plasmids by fusing either an adenine or cytosine deaminase domain to catalytically inactive LbCas12a-RVQ or Flex-Cas12a, to generate four base editor constructs: LbCas12a-RVQ-ABE, LbCas12a-RVQ-CBE, Flex-Cas12a-ABE, and Flex-Cas12a-CBE. We also designed crRNAs to enable targeting of four endogenous genomic loci for ABE and CBE, respectively. Following plasmid transfection of these BEs into HEK293T cells, NGS data revealed that Flex-Cas12a-derived BEs achieved comparable editing efficiencies to their LbCas12a-RVQ counterparts at canonical 5′-TTTV-3′ PAM sites, but exhibited two- to three-fold higher editing efficiencies at sites with noncanonical PAMs (Fig. 5C and Supplementary Fig. S6A and B). These results are consistent with our nuclease-based editing data and demonstrate that Flex-Cas12a not only matches the activities of LbCas12a-RVQ at sites with canonical PAMs but also enables efficient editing at noncanonical PAMs, broadening its genome-editing potential in mammalian cells. Notably, low-level base editing activity was observed for LbCas12a-RVQ at several noncanonical PAM sites. This residual editing may result from transient or partial DNA binding that permits deamination in the absence of complete R-loop formation [41, 54]. Such off-target deamination events have been previously reported [55] and are thought to arise from the inherent activity of deaminase domains acting on accessible single-stranded regions, even when cleavage is inefficient or PAM recognition is suboptimal. In addition, we note that the difference in base editing activity (Fig. 5C and Supplementary Fig. S6) between Flex-Cas12a and RVQ appears smaller than the difference observed in their nuclease editing efficiencies (Fig. 5A and B). This may be due in part to delivery-dependent effects: base editing experiments were performed via plasmid transfection, which results in prolonged intracellular expression. Under these conditions, variants such as RVQ may partially compensate for reduced binding kinetics on noncanonical PAMs, narrowing the performance gap with Flex-Cas12a. This trend is consistent with our observations in plasmid-based nuclease assays (Supplementary Fig. S4) and reinforces the importance of delivery context when evaluating editing outcomes.

Taken together, these findings establish Flex-Cas12a as a versatile genome-editing tool that enables modification of previously unreachable genomic loci, providing a robust new platform for both research and therapeutic applications.

## Discussion

Directed evolution has been demonstrated as a powerful strategy for tailoring CRISPR–Cas enzymes to enhance catalytic activity [3, 13, 15, 31], alter or expand the targeting scope [33, 56, 57], or improve specificity [35, 58, 59]. In this study, we used an unbiased directed evolution approach to identify seven PAM-relaxed LbCas12a variants with expanded DNA target recognition and genome-editing capabilities. Through further rational engineering, we developed an optimized variant, Flex-Cas12a, as an effective RNA-guided endonuclease with expanded PAM recognition and minimal off-target effects across various delivery platforms (Figs 4 and 5, and Supplementary Figs S4 and S5). Flex-Cas12a recognizes a wide spectrum of 5′-NYHV-3′ PAMs while retaining robust activity with the canonical 5′-TTTV-3′ PAM. This expands the targetable human genome from approximately 1% to over 25%, representing a substantial improvement. Our findings build on and extend recent advances in engineering Cas12a variants with either enhanced catalytic activity (e.g. enAsCas12a, opAsCas12a, AsCas12a-Ultra, iCas12a, and LbCas12a-RVQ) [3, 13, 15, 16, 60] or relaxed PAM requirements (e.g. AsCas12a-RR/RVR and LbCas12a-RVR/RVRR) [17, 47]. However, even the most PAM-relaxed enzymes among these are theoretically limited to targeting only ∼6% of the genome. In contrast, Flex-Cas12a substantially expands this limit, making it a valuable addition to the CRISPR toolkit for applications requiring greater target flexibility.

To understand the molecular mechanism of Flex-Cas12a’s expanded PAM recognition, we mapped all six mutations in this protein onto the crystal structure of the LbCas12a–crRNA–DNA ternary complex (PDB: 5XUS). We hypothesize that the D535G substitution alters interactions between the protein and DNA backbone in the PAM region (Fig. 1B, bottom panel) and potentially broadens its PAM recognition. The introduced activity-enhancing mutations, including G146R, R182V, and E795Q [16], are positioned distal to the PAM-binding interface and hence are likely to promote overall catalytic efficiency rather than alter PAM recognition, consistent with our experimental findings (Fig. 3) [16]. Taken together, these structural insights support the conclusion that Flex-Cas12a maintains high enzymatic activity while accommodating a significantly broader PAM repertoire.

Preliminary biochemical data (Fig. 3A and B, and Supplementary Fig. S7) further suggest that Flex-Cas12a avoids off-target entrapment observed for PAM-relaxed Cas9 variants [21, 23], preserving specificity despite its increased target range. This highlights Flex-Cas12a’s potential as a more versatile yet precise genome-editing platform. Future biophysical analysis of these variants may help uncover the energetic landscape governing target DNA binding and cleavage by Flex-Cas12a, ultimately guiding further improvements in CRISPR enzyme design. Additional strategies may further enhance Flex-Cas12a’s performance. For instance, combinatorial engineering with optimized crRNA scaffolds [61] through modifications to the stem-loop or guide sequence has been shown to improve editing efficiency and expand targeting capabilities in other Cas12a variants [62, 63] or different CRISPR enzymes [64]. Furthermore, integrating negative selection modalities into directed evolution, where off-target cleavage leads to bacterial lethality, could complement our current positive selection approach and help evolve Cas12a variants with improved specificity, as previously demonstrated for Cas9 [35].

In summary, Flex-Cas12a represents a powerful genome editor combining broad PAM compatibility, high on-target activity, and low off-target effects. Its application across multiple editing platforms, including nuclease, base editing, and multiplexed targeting, positions it as a next-generation Cas12a variant with broad translational potential.

## Supplementary Material

gkaf649_Supplemental_Files

## Data Availability

Next generation sequencing data have been deposited to SRA (https://www.ncbi.nlm.nih.gov/sra) under accession number PRJNA1278891. All the plasmid maps will be deposited to Addgene (https://www.addgene.org/) upon publication.

## References

[1] Zetsche B, Gootenberg JS, Abudayyeh OO et al. Cpf1 is a single RNA-guided endonuclease of a class 2 CRISPR–Cas system. Cell. 2015; 163:759–71. 10.1016/j.cell.2015.09.038. 26422227 PMC4638220

[2] Banakar R, Schubert M, Kurgan G et al. Efficiency, specificity and temperature sensitivity of Cas9 and Cas12a RNPs for DNA-free genome editing in plants. Front Genome Ed. 2021; 3:760820 10.3389/fgeed.2021.760820. 35098208 PMC8790294

[3] Zhang L, Zuris JA, Viswanathan R et al. AsCas12a ultra nuclease facilitates the rapid generation of therapeutic cell medicines. Nat Commun. 2021; 12:3908 10.1038/s41467-021-24017-8. 34162850 PMC8222333

[4] Zhang R, Chai N, Liu T et al. The type V effectors for CRISPR/Cas-mediated genome engineering in plants. Biotechnol Adv. 2024; 74:108382 10.1016/j.biotechadv.2024.108382. 38801866

[5] Fonfara I, Richter H, Bratovič M et al. The CRISPR-associated DNA-cleaving enzyme Cpf1 also processes precursor CRISPR RNA. Nature. 2016; 532:517–21. 10.1038/nature17945. 27096362

[6] Yamano T, Nishimasu H, Zetsche B et al. Crystal structure of Cpf1 in complex with guide RNA and target DNA. Cell. 2016; 165:949–62. 10.1016/j.cell.2016.04.003. 27114038 PMC4899970

[7] Swarts DC, van der Oost J, Jinek M Structural basis for guide RNA processing and seed-dependent DNA targeting by CRISPR–Cas12a. Mol Cell. 2017; 66:221–33. 10.1016/j.molcel.2017.03.016. 28431230 PMC6879319

[8] Wang JY, Doudna JA CRISPR technology: a decade of genome editing is only the beginning. Science. 2023; 379:eadd8643 10.1126/science.add8643. 36656942

[9] Pacesa M, Pelea O, Jinek M Past, present, and future of CRISPR genome editing technologies. Cell. 2024; 187:1076–100. 10.1016/j.cell.2024.01.042. 38428389

[10] Kim H, Kim S-T, Ryu J et al. CRISPR/Cpf1-mediated DNA-free plant genome editing. Nat Commun. 2017; 8:14406 10.1038/ncomms14406. 28205546 PMC5316869

[11] Tang X, Lowder LG, Zhang T et al. A CRISPR–Cpf1 system for efficient genome editing and transcriptional repression in plants. Nat Plants. 2017; 3:17018. 28211909 10.1038/nplants.2017.18

[12] Zetsche B, Heidenreich M, Mohanraju P et al. Multiplex gene editing by CRISPR–Cpf1 using a single crRNA array. Nat Biotechnol. 2017; 35:31–4. 10.1038/nbt.3737. 27918548 PMC5225075

[13] Kleinstiver BP, Sousa AA, Walton RT et al. Engineered CRISPR–Cas12a variants with increased activities and improved targeting ranges for gene, epigenetic and base editing. Nat Biotechnol. 2019; 37:276–82. 10.1038/s41587-018-0011-0. 30742127 PMC6401248

[14] Guo LY, Bian J, Davis AE et al. Multiplexed genome regulation *in vivo* with hyper-efficient Cas12a. Nat Cell Biol. 2022; 24:590–600. 10.1038/s41556-022-00870-7. 35414015 PMC9035114

[15] Ma E, Chen K, Shi H et al. Improved genome editing by an engineered CRISPR–Cas12a. Nucleic Acids Res. 2022; 50:12689–701. 10.1093/nar/gkac1192. 36537251 PMC9825149

[16] Zhang L, Li G, Zhang Y et al. Boosting genome editing efficiency in human cells and plants with novel LbCas12a variants. Genome Biol. 2023; 24:102 10.1186/s13059-023-02929-6. 37122009 PMC10150537

[17] Tóth E, Varga É, Kulcsár PI et al. Improved LbCas12a variants with altered PAM specificities further broaden the genome targeting range of Cas12a nucleases. Nucleic Acids Res. 2020; 48:3722–33. 10.1093/nar/gkaa110. 32107556 PMC7144938

[18] Tóth E, Czene BC, Kulcsár PI et al. Mb- and FnCpf1 nucleases are active in mammalian cells: activities and PAM preferences of four wild-type Cpf1 nucleases and of their altered PAM specificity variants. Nucleic Acids Res. 2018; 46:10272–85. 30239882 10.1093/nar/gky815 PMC6212782

[19] Walton RT, Christie KA, Whittaker MN et al. Unconstrained genome targeting with near-PAMless engineered CRISPR–Cas9 variants. Science. 2020; 368:290–6. 10.1126/science.aba8853. 32217751 PMC7297043

[20] Choi E, Hwang H-Y, Kwon E et al. Expanded targeting scope of LbCas12a variants allows editing of multiple oncogenic mutations. Mol Ther Nucleic Acids. 2022; 30:131–42. 10.1016/j.omtn.2022.09.005. 36250202 PMC9535386

[21] Hibshman GN, Bravo JPK, Hooper MM et al. Unraveling the mechanisms of PAMless DNA interrogation by SpRY-Cas9. Nat Commun. 2024; 15:3663 10.1038/s41467-024-47830-3. 38688943 PMC11061278

[22] Olivi L, Bagchus C, Pool V et al. Live-cell imaging reveals the trade-off between target search flexibility and efficiency for Cas9 and Cas12a. Nucleic Acids Res. 2024; 52:5241–56. 10.1093/nar/gkae283. 38647045 PMC11109954

[23] Shi H, Al-Sayyad N, Wasko KM et al. Rapid two-step target capture ensures efficient CRISPR–Cas9-guided genome editing. Mol Cell. 2025; 85:1730–42. 10.1016/j.molcel.2025.03.024. 40273916 PMC12258621

[24] Kleinstiver BP, Tsai SQ, Prew MS et al. Genome-wide specificities of CRISPR–Cas Cpf1 nucleases in human cells. Nat Biotechnol. 2016; 34:869–74. 10.1038/nbt.3620. 27347757 PMC4980201

[25] Moreno-Mateos MA, Fernandez JP, Rouet R et al. CRISPR–Cpf1 mediates efficient homology-directed repair and temperature-controlled genome editing. Nat Commun. 2017; 8:2024 10.1038/s41467-017-01836-2. 29222508 PMC5722943

[26] Malzahn AA, Tang X, Lee K et al. Application of CRISPR–Cas12a temperature sensitivity for improved genome editing in rice, maize, and *Arabidopsis*. BMC Biol. 2019; 17:9 10.1186/s12915-019-0629-5. 30704461 PMC6357469

[27] Zhang Y, Malzahn AA, Sretenovic S et al. The emerging and uncultivated potential of CRISPR technology in plant science. Nat Plants. 2019; 5:778–94. 10.1038/s41477-019-0461-5. 31308503

[28] Zhang Y, Wu Y, Li G et al. Genome-wide investigation of multiplexed CRISPR–Cas12a-mediated editing in rice. Plant Genome. 2023; 16:e20266 10.1002/tpg2.20266. 36177842 PMC12806924

[29] Swarts DC, Jinek M Cas9 versus Cas12a/Cpf1: structure–function comparisons and implications for genome editing. Wiley Interdiscip Rev RNA. 2018; 9:e1481 10.1002/wrna.1481. 29790280

[30] Eggers AR, Chen K, Soczek KM et al. Rapid DNA unwinding accelerates genome editing by engineered CRISPR–Cas9. Cell. 2024; 187:3249–61. 10.1016/j.cell.2024.04.031. 38781968 PMC11658890

[31] Chen K, Han H, Zhao S et al. Lung and liver editing by lipid nanoparticle delivery of a stable CRISPR–Cas9 ribonucleoprotein. Nat Biotechnol. 2024; 10.1038/s41587-024-02437-3. PMC12000389 39415058

[32] Chen Z, Zhao H A highly sensitive selection method for directed evolution of homing endonucleases. Nucleic Acids Res. 2005; 33:e154 10.1093/nar/gni148. 16214805 PMC1253837

[33] Kleinstiver BP, Prew MS, Tsai SQ et al. Engineered CRISPR–Cas9 nucleases with altered PAM specificities. Nature. 2015; 523:481–5. 10.1038/nature14592. 26098369 PMC4540238

[34] Hu JH, Miller SM, Geurts MH et al. Evolved Cas9 variants with broad PAM compatibility and high DNA specificity. Nature. 2018; 556:57–63. 10.1038/nature26155. 29512652 PMC5951633

[35] Lee JK, Jeong E, Lee J et al. Directed evolution of CRISPR–Cas9 to increase its specificity. Nat Commun. 2018; 9:3048 10.1038/s41467-018-05477-x. 30082838 PMC6078992

[36] Walton RT, Hsu JY, Joung JK et al. Scalable characterization of the PAM requirements of CRISPR–Cas enzymes using HT-PAMDA. Nat Protoc. 2021; 16:1511–47. 10.1038/s41596-020-00465-2. 33547443 PMC8063866

[37] Chen F, Lian M, Ma B et al. Multiplexed base editing through Cas12a variant-mediated cytosine and adenine base editors. Commun Biol. 2022; 5:1163 10.1038/s42003-022-04152-8. 36323848 PMC9630288

[38] Bae S, Park J, Kim J-S Cas-OFFinder: a fast and versatile algorithm that searches for potential off-target sites of Cas9 RNA-guided endonucleases. Bioinformatics. 2014; 30:1473–5. 10.1093/bioinformatics/btu048. 24463181 PMC4016707

[39] Clement K, Rees H, Canver MC et al. CRISPResso2 provides accurate and rapid genome editing sequence analysis. Nat Biotechnol. 2019; 37:224–6. 10.1038/s41587-019-0032-3. 30809026 PMC6533916

[40] Yamano T, Zetsche B, Ishitani R et al. Structural basis for the canonical and non-canonical PAM recognition by CRISPR–Cpf1. Mol Cell. 2017; 67:633–45. 10.1016/j.molcel.2017.06.035. 28781234 PMC5957536

[41] Strohkendl I, Saha A, Moy C et al. Cas12a domain flexibility guides R-loop formation and forces RuvC resetting. Mol Cell. 2024; 84:2717–31. 10.1016/j.molcel.2024.06.007. 38955179 PMC11283365

[42] Madisen L, Zwingman TA, Sunkin SM et al. A robust and high-throughput Cre reporting and characterization system for the whole mouse brain. Nat Neurosci. 2010; 13:133–40. 10.1038/nn.2467. 20023653 PMC2840225

[43] Staahl BT, Benekareddy M, Coulon-Bainier C et al. Efficient genome editing in the mouse brain by local delivery of engineered Cas9 ribonucleoprotein complexes. Nat Biotechnol. 2017; 35:431–4. 10.1038/nbt.3806. 28191903 PMC6649674

[44] Li S-Y, Cheng Q-X, Liu J-K et al. CRISPR–Cas12a has both *cis*- and *trans*-cleavage activities on single-stranded DNA. Cell Res. 2018; 28:491–3. 10.1038/s41422-018-0022-x. 29531313 PMC5939048

[45] Chen JS, Ma E, Harrington LB et al. CRISPR–Cas12a target binding unleashes indiscriminate single-stranded DNase activity. Science. 2018; 360:436–9. 10.1126/science.aar6245. 29449511 PMC6628903

[46] Fu Y, Foden JA, Khayter C et al. High frequency off-target mutagenesis induced by CRISPR–Cas nucleases in human cells. Nat Biotechnol. 2013; 31:822–6. 10.1038/nbt.2623. 23792628 PMC3773023

[47] Gao L, Cox DBT, Yan WX et al. Engineered Cpf1 variants with altered PAM specificities increase genome targeting range. Nat Biotechnol. 2017; 35:789–92. 10.1038/nbt.3900. 28581492 PMC5548640

[48] Nishimasu H, Yamano T, Gao L et al. Structural basis for the altered PAM recognition by engineered CRISPR–Cpf1. Mol Cell. 2017; 67:139–147. 10.1016/j.molcel.2017.04.019. 28595896 PMC5957533

[49] Wang H, Liu B, Wei J Beta2-microglobulin (B2M) in cancer immunotherapies: biological function, resistance and remedy. Cancer Lett. 2021; 517:96–104. 10.1016/j.canlet.2021.06.008. 34129878

[50] Hamilton JR, Tsuchida CA, Nguyen DN et al. Targeted delivery of CRISPR–Cas9 and transgenes enables complex immune cell engineering. Cell Rep. 2021; 35:109207 10.1016/j.celrep.2021.109207. 34077734 PMC8236216

[51] Stahl EC, Sabo JK, Kang MH et al. Genome editing in the mouse brain with minimally immunogenic Cas9 RNPs. Mol Ther. 2023; 31:2422–38. 10.1016/j.ymthe.2023.06.019. 37403358 PMC10422012

[52] Foss DV, Muldoon JJ, Nguyen DN et al. Peptide-mediated delivery of CRISPR enzymes for the efficient editing of primary human lymphocytes. Nat Biomed Eng. 2023; 7:647–60. 10.1038/s41551-023-01032-2. 37147433 PMC10129304

[53] Zhang Z, Baxter AE, Ren D et al. Efficient engineering of human and mouse primary cells using peptide-assisted genome editing. Nat Biotechnol. 2024; 42:305–15. 10.1038/s41587-023-01756-1. 37095348 PMC11230135

[54] Lapinaite A, Knott GJ, Palumbo CM et al. DNA capture by a CRISPR–Cas9-guided adenine base editor. Science. 2020; 369:566–71. 10.1126/science.abb1390. 32732424 PMC8598131

[55] Yang L, Briggs AW, Chew WL et al. Engineering and optimising deaminase fusions for genome editing. Nat Commun. 2016; 7:13330 10.1038/ncomms13330. 27804970 PMC5097136

[56] Miller SM, Wang T, Randolph PB et al. Continuous evolution of SpCas9 variants compatible with non-G PAMs. Nat Biotechnol. 2020; 38:471–81. 10.1038/s41587-020-0412-8. 32042170 PMC7145744

[57] Huang TP, Heins ZJ, Miller SM et al. High-throughput continuous evolution of compact Cas9 variants targeting single-nucleotide-pyrimidine PAMs. Nat Biotechnol. 2023; 41:96–107. 10.1038/s41587-022-01410-2. 36076084 PMC9849140

[58] Casini A, Olivieri M, Petris G et al. A highly specific SpCas9 variant is identified by *in vivo* screening in yeast. Nat Biotechnol. 2018; 36:265–71. 10.1038/nbt.4066. 29431739 PMC6066108

[59] Goldberg GW, Spencer JM, Giganti DO et al. Engineered dual selection for directed evolution of SpCas9 PAM specificity. Nat Commun. 2021; 12:349 10.1038/s41467-020-20650-x. 33441553 PMC7807044

[60] Gier RA, Budinich KA, Evitt NH et al. High-performance CRISPR–Cas12a genome editing for combinatorial genetic screening. Nat Commun. 2020; 11:3455 10.1038/s41467-020-17209-1. 32661245 PMC7359328

[61] Dong C, Gou Y, Lian J SgRNA engineering for improved genome editing and expanded functional assays. Curr Opin Biotechnol. 2022; 75:102697 10.1016/j.copbio.2022.102697. 35217295

[62] Teng F, Li J, Cui T et al. Enhanced mammalian genome editing by new Cas12a orthologs with optimized crRNA scaffolds. Genome Biol. 2019; 20:15 10.1186/s13059-019-1620-8. 30717767 PMC6362571

[63] Jedrzejczyk DJ, Poulsen LD, Mohr M et al. CRISPR–Cas12a nucleases function with structurally engineered crRNAs: synThetic trAcrRNA. Sci Rep. 2022; 12:12193 10.1038/s41598-022-15388-z. 35842430 PMC9288538

[64] Kocak DD, Josephs EA, Bhandarkar V et al. Increasing the specificity of CRISPR systems with engineered RNA secondary structures. Nat Biotechnol. 2019; 37:657–66. 10.1038/s41587-019-0095-1. 30988504 PMC6626619

