# Supplementary material for: Directed evolution expands CRISPR–Cas12a genome-editing capacity
Source: Nucleic Acids Res. 2025 Jul 16;53(13):gkaf649. doi: 10.1093/nar/gkaf649 (PMC12266133; doi:10.1093/nar/gkaf649)
Supplement: gkaf649_Supplemental_Files [file gkaf649_supplemental_files.zip › Rev_Ma_et_al_FlexCas12a_final_supplementary_figures.pdf]

Supplementary Data

Directed evolution expands CRISPR-Cas12a genome-editing capacity

Enbo Ma[1,2*], Kai Chen[1,2*], Honglue Shi[1,3*], Kevin M. Wasko[1,2], Isabel Esain-Garcia[1,4], Marena I. Trinidad[1,4], Kaihong Zhou[3], Jinjuan Ye[3], Jennifer A. Doudna[1-9†]

[1]Innovative Genomics Institute, University of California, Berkeley, Berkeley, CA 94720, USA;

[2]Department of Molecular and Cell Biology, University of California, Berkeley, Berkeley, CA 94720, USA;

[3]Howard Hughes Medical Institute, University of California, Berkeley, Berkeley, CA 94720, USA;

[4]California Institute for Quantitative Biosciences (QB3), University of California, Berkeley, Berkeley, CA 94720, USA;

[5]Molecular Biophysics and Integrated Bioimaging Division, Lawrence Berkeley National Laboratory, Berkeley, CA 94720, USA;

[6]Li Ka Shing Center for Genomic Engineering, University of California, Berkeley, Berkeley, CA 94720, USA;

[7]Department of Chemistry, University of California, Berkeley, Berkeley, CA 94720, USA;

[8]Gladstone Institute of Data Science and Biotechnology, San Francisco, CA 94158, USA;

[9]Gladstone-UCSF Institute of Genomic Immunology, San Francisco, CA 94158, USA

*These authors contributed equally to this work.

†Correspondence: doudna@berkeley.edu

Supplementary Figures

A

crRNA

Name	Guide sequences
crRNA1	UUCAUCCCCGAUAUGCACCACCGG
crRNA2	UCCCGUGAACUUUACCCGGUGGUG
crRNA3	UAUCGGGGAUGAAAGCUGGCGCAU
crRNA4	AUAAAGUCUCCCGUGAACUUUACC

Target

Name	Target sequences in *ccdB* gene (PAMs in bold)
T1	**AGCT**TTCATCCCCGATATGCACCACCGG
T2	**AGTC**TCCCGTGAACTTTACCCGGTGGTG
T3	**TGCA**TATCGGGGATGAAAGCTGGCGCAT
T4	**TCAG**ATAAAGTCTCCCGTGAACTTTACC

B

Seven selected mutants

crRNA	Mutant	Mutations
crRNA2	Lbm1	K538N, N590Y
	Lbm2	Q529R, K538N, E610G
	Lbm3	Q529R, K538N, K753R, E754A
	Lbm4	K538N, Y553F, D691G, K752R, P799R
crRNA3	Lbm5	K538N, Y549C, E743K
crRNA4	Lbm6	D535G, S551F, D665N
	Lbm7	D535G, D573G, N628S, I765T

D

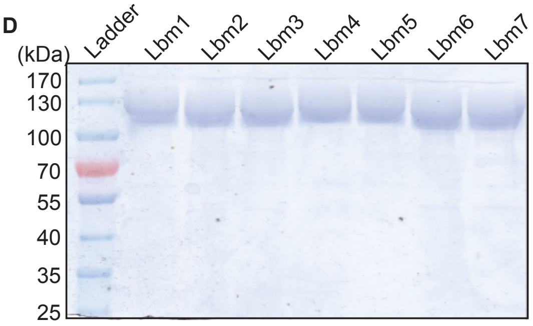

C

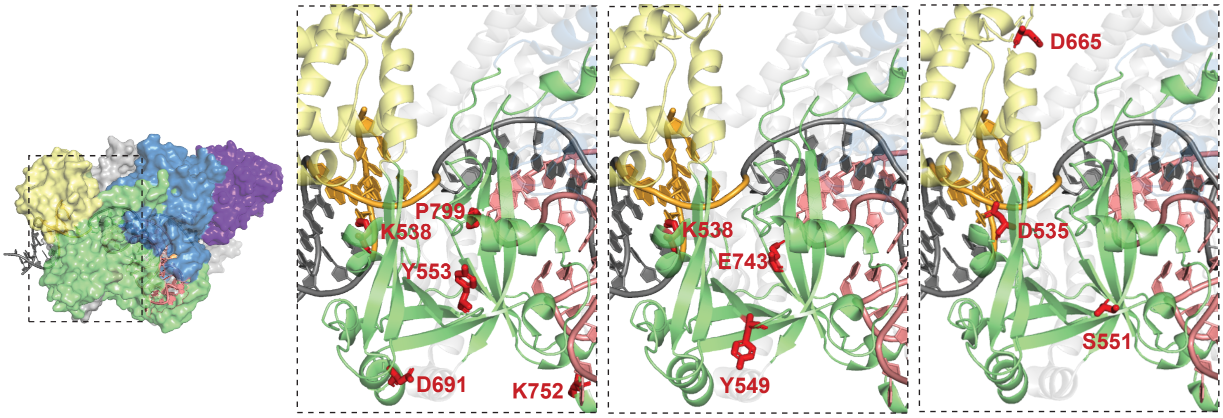

LbCas12a structure **Lbm4** **Lbm5** **Lbm6**

E

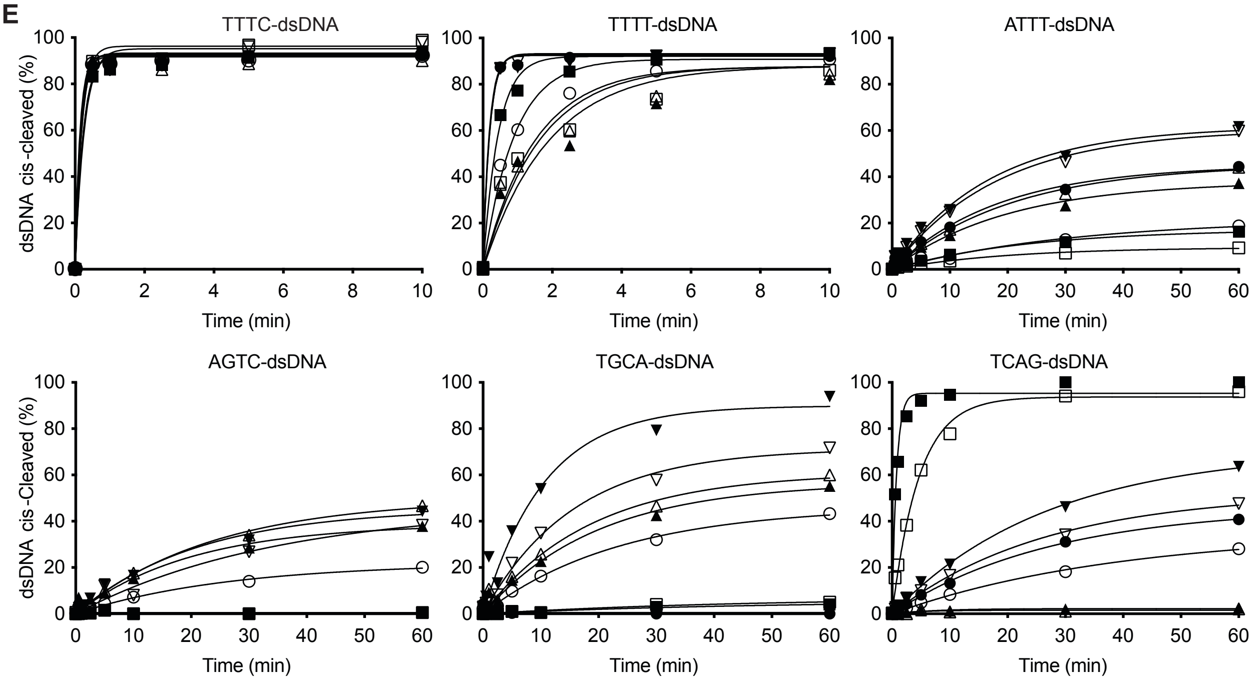

K_{obs} of each protein with different PAM-dsDNAs (min^{-1})

Protein	Different PAM-dsDNAs					
	TTTC	TTTT	ATTT	AGTC	TGCA	TCAG
wt	5.643	5.637	0.055	0.778	nd	0.037
Lbm1	6.577	1.119	0.032	0.038	0.042	0.026
Lbm2	6.653	0.527	0.050	0.050	0.051	0.128
Lbm3	7.323	0.619	0.049	0.042	0.054	0.203
Lbm4	4.244	5.281	0.059	0.047	0.094	0.037
Lbm5	3.788	5.243	0.055	0.029	0.064	0.038
Lbm6	4.302	2.296	0.043	0.778	0.021	1.294
Lbm7	5.142	0.673	0.050	0.778	0.022	0.213

F

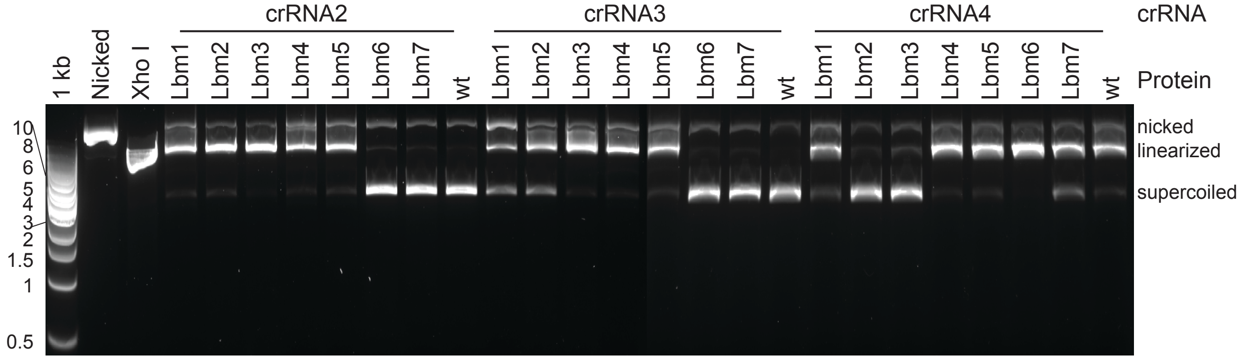

G

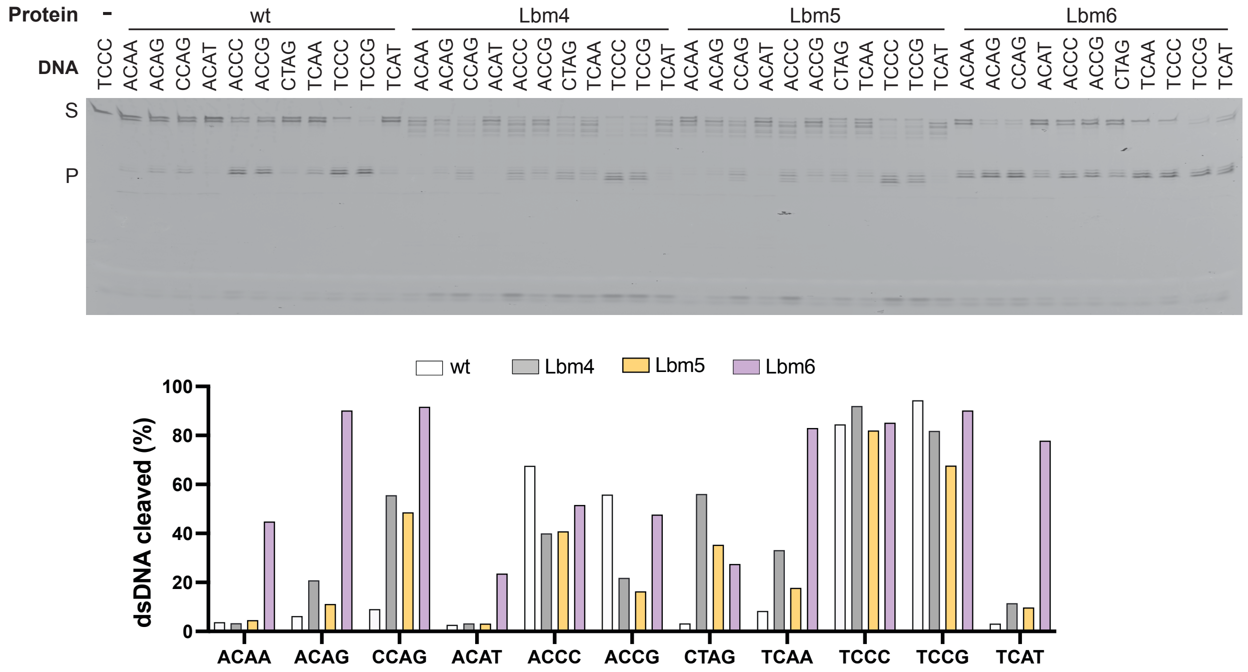

Supplementary Figure S1. Generation and analysis of PAM-relaxed variants.

A. Target sequences for directed evolution. Left panel shows the target sequences of four crRNAs (crRNA1 to 4) and right panel shows four *ccdB* target DNA sequences (presented as non-target strands) with randomly selected non-canonical PAMs (AGCT, AGTC, TGCA, or TCAG). **B.** Mutations in PAM-relaxed LbCas12a variants. Residues highlighted in bold indicate that they are localized within the PAM-interacting domain (PI). **C.** Structural presentation of the LbCas12a-dsDNA-crRNA ternary complex (PDB: 5XUS), highlighting mutation sites in Lbm4, Lbm5, and Lbm6. The PI domain is colored in yellow, and the WED domains are in green, PAM sequence is shown in brown, and all mutated residues are labeled in red. **D.** An SDS-PAGE gel image of the purified seven variants Lbm1-7. **E.** *In vitro* kinetic analysis of *cis*-cleavage activity. Cleavage efficiencies of wild-type (wt) and seven variants (Lbm1 to Lbm7) are shown. Data points represent the mean of two independent experiments. Target DNAs (derivatives of T0, listed in Supplementary Table S1) used in these assays are the same except for PAM sequences. TTTC is a canonical PAM, while others represent non-canonical PAMs. Each PAM sequence of the DNA substrate is listed at the top of each panel. K_{obs} of each LbCas12a variant is listed. **F.**

Plasmid cleavage assays of *ccdB*-containing plasmid DNA. Each designed crRNA targets a DNA sequence with a non-canonical PAM (crRNA2: AGTC, crRNA3: TGCA and crRNA4: TCAG). Each crRNA was tested against the seven Lbm1-7 variants as well as wild-type LbCas12a (wt). **G.** *In vitro* cleavage assays with various synthetic DNA substrates that are derived from DNA T0 with different non-canonical PAM sequences. The corresponding PAM sequences are listed on top of the gel image or on the X-axis. Top panel presents a representative gel image. Lower panel quantifies cleavage efficiencies across eleven target DNAs by wt, Lbm4, Lbm5 and Lbm6, respectively. S represents the substrate, and P represents the cleavage products.

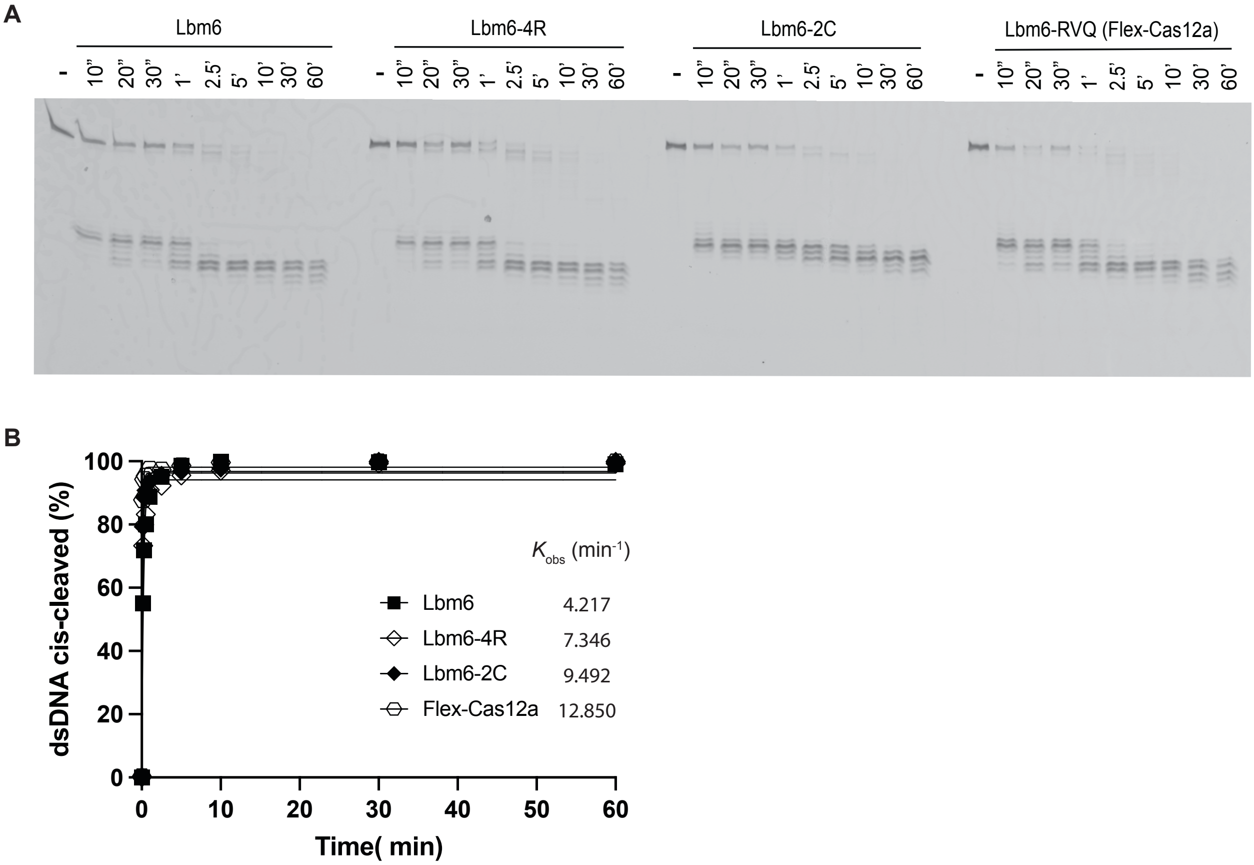

C.

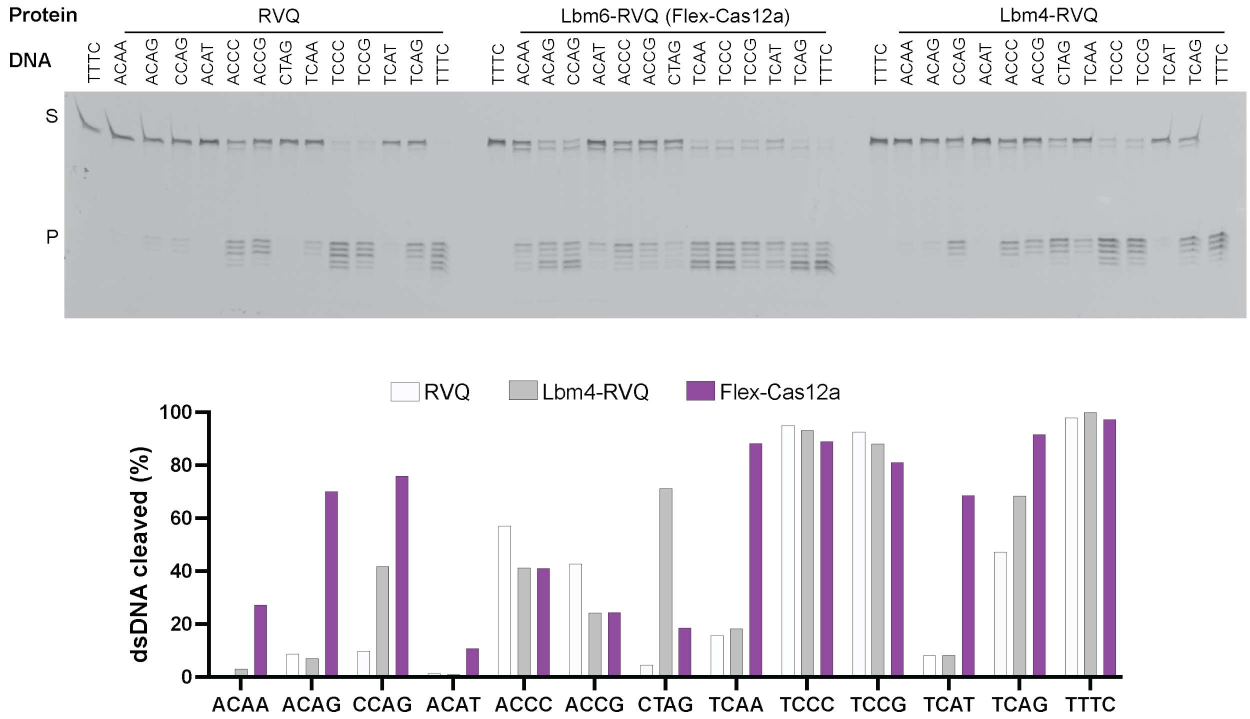

Supplementary Figure S2. DNA cleavage assays by Lbm6 and its derivatives

A. A gel image of *in vitro* cleavages by Lbm6 and its derivatives of Lbm6-4R, Lbm6-2C, and Lbm6-RVQ. Target DNA used in this assay is DNA T0 (listed in Supplementary Table S1) with a PAM of 5'-TTTC-3'). **B.** Quantification of cleavage efficiencies of each variant over time. The cleavage results indicate that Lbm6-RVQ exhibits the highest activity and is renamed Flex-Cas12a from now on. K_{obs} of each LbCas12a variant is listed. **C.** *In vitro* cleavage assays of DNA substrates bearing different PAMs. Lbm4-RVQ, Flex-Cas12a and LbCas12a-RVQ (RVQ) were assessed. Upper panel shows a gel image of DNA cleavage reactions. Lower panel quantifies cleavage efficiencies for thirteen target DNAs with each protein. Target DNAs used in these assays are DNA T0 with different PAMs which are listed on top of the gel image or on the X-axis. TTTC is a canonical PAM, while others represent non-canonical PAMs. S represents the substrate, and P represents cleavage products.

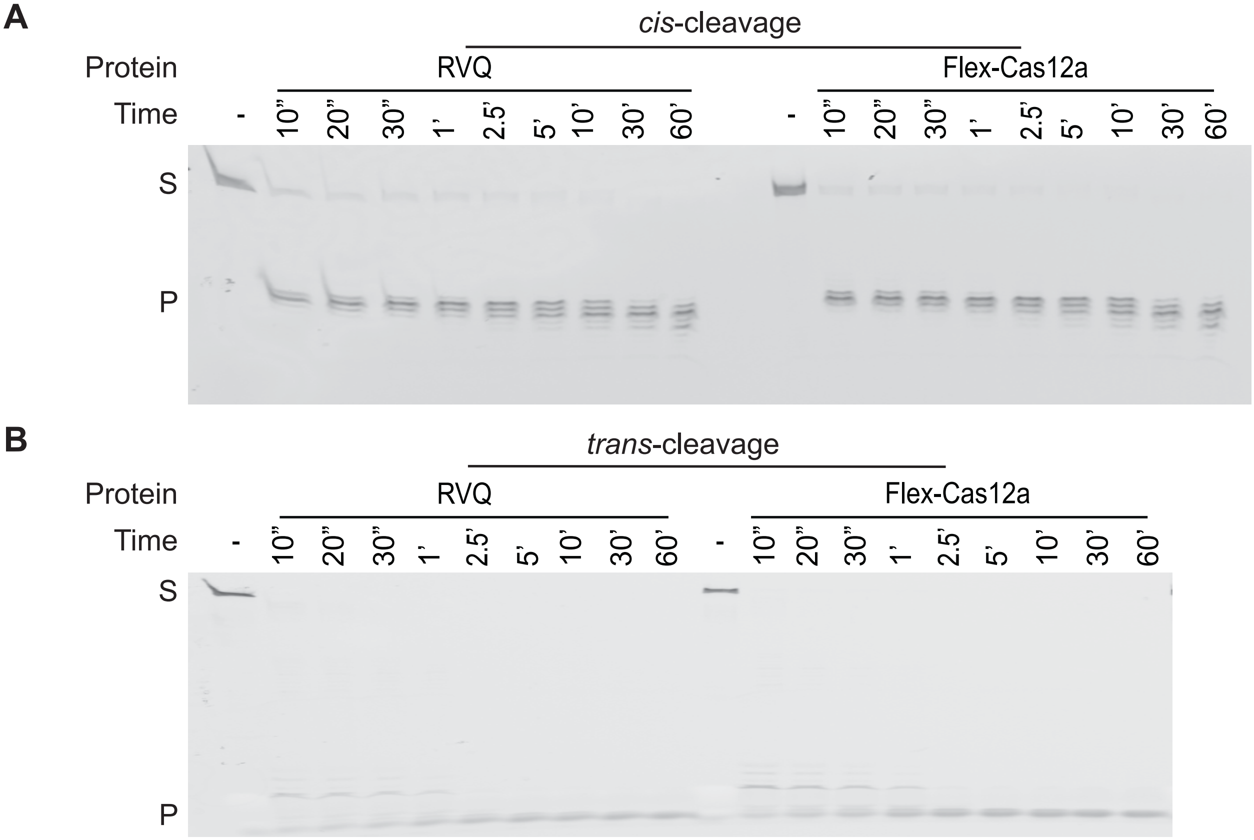

Supplementary Figure S3. Comparison of DNA cleavage activities of Flex-Cas12a to LbCas12a-RVQ (RVQ).

A. The gel image of *cis*-cleavage assays. DNA substrate used in assay is DNA T0 with a canonical PAM of 5'-TTTC-3'. **B.** The gel image of *trans*-cleavage assays. In this assay, 45 nM unlabeled target dsDNA T0 with a canonical PAM of 5'-TTTC-3' was incubated with LbCas12a RNPs for 30 min at 37°C before addition of a labeled random ssDNA (no homology with the target DNAs or crRNAs, listed in Supplementary Table S1).

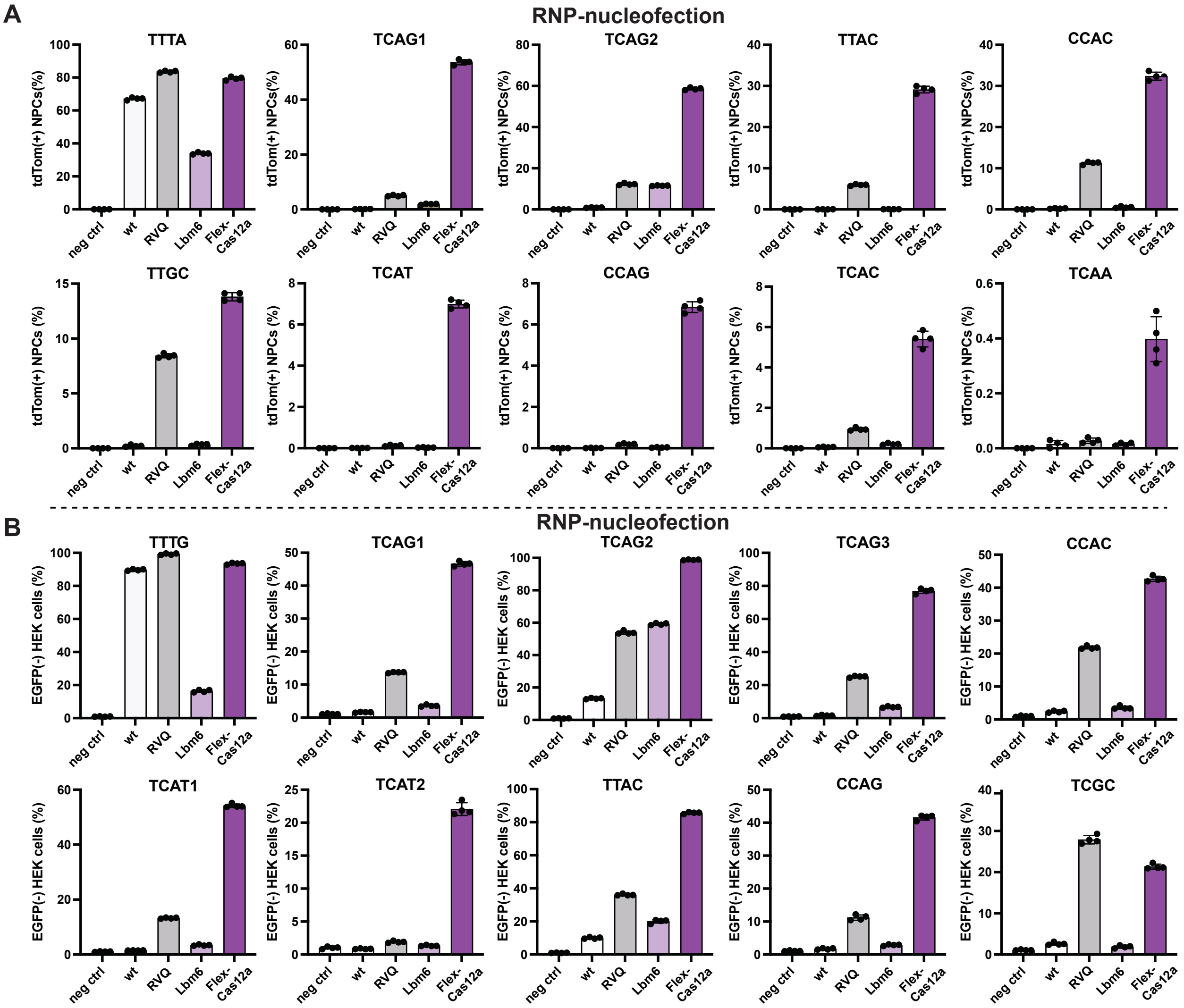
A
RNP-nucleofection
TTTA
TCAG1
TCAG2
TTAC
CCAC
TTGC
TCAT
CCAG
TCAC
TCAA
tdTom(+) NPCs(%)
tdTom(+) NPCs (%)
neg ctrl
wt
RVQ
Lbm6
Flex-Cas12a
B
RNP-nucleofection
TTTG
TCAG1
TCAG2
TCAG3
CCAC
TCAT1
TCAT2
TTAC
CCAG
TCGC
EGFP(-) HEK cells (%)

C **Plasmid transfection (150 ng)**

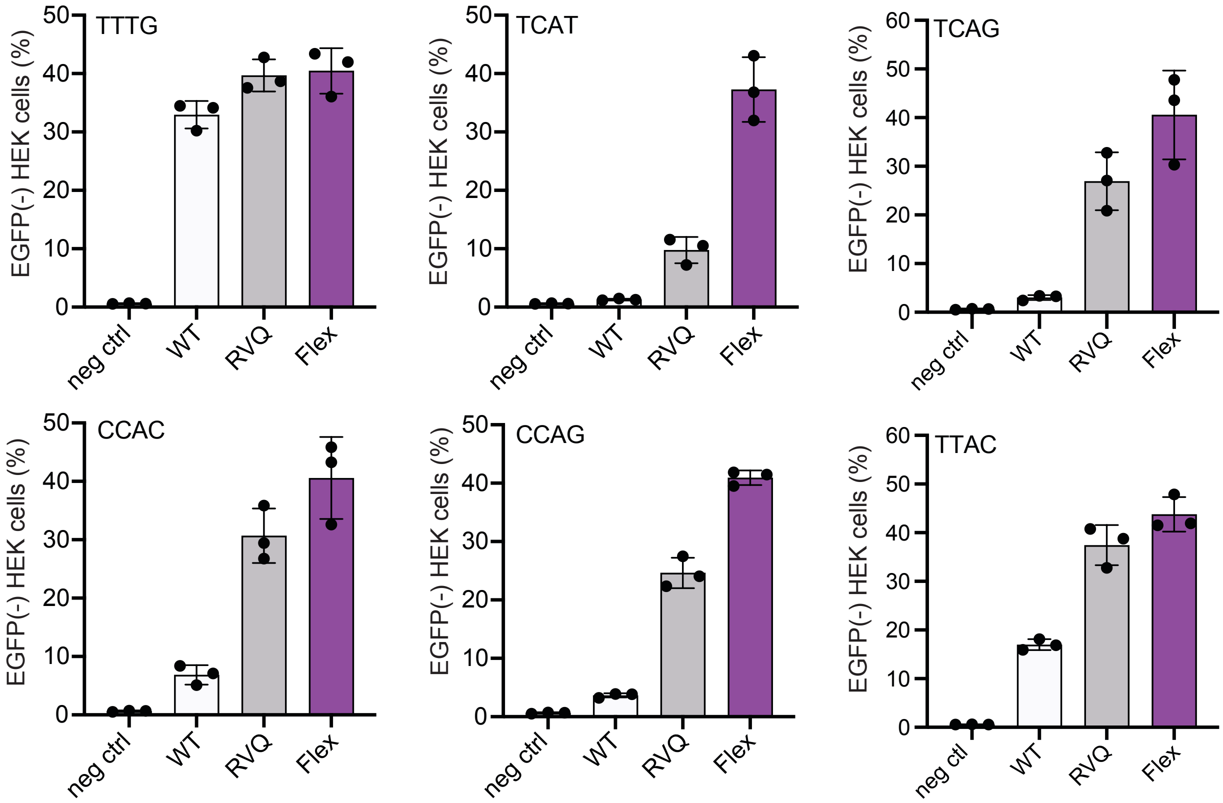

D **Plasmid transfection (200 ng)**

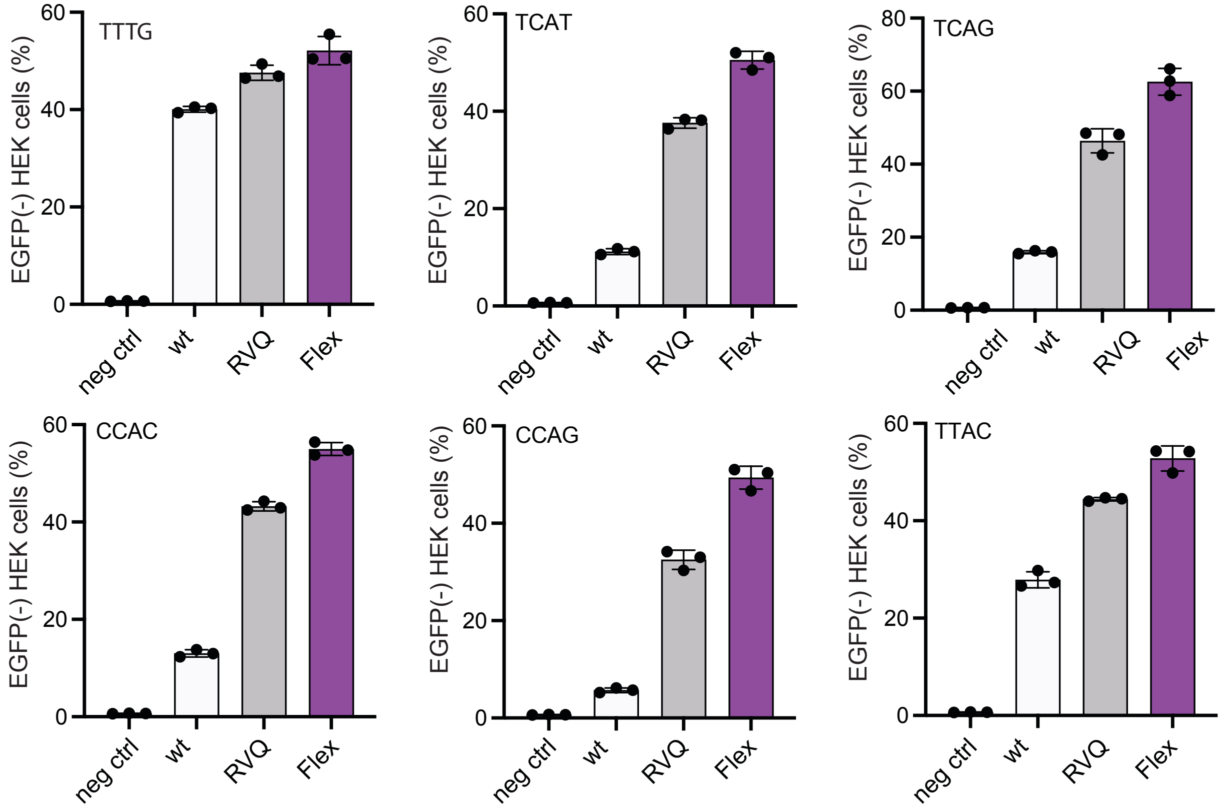

E

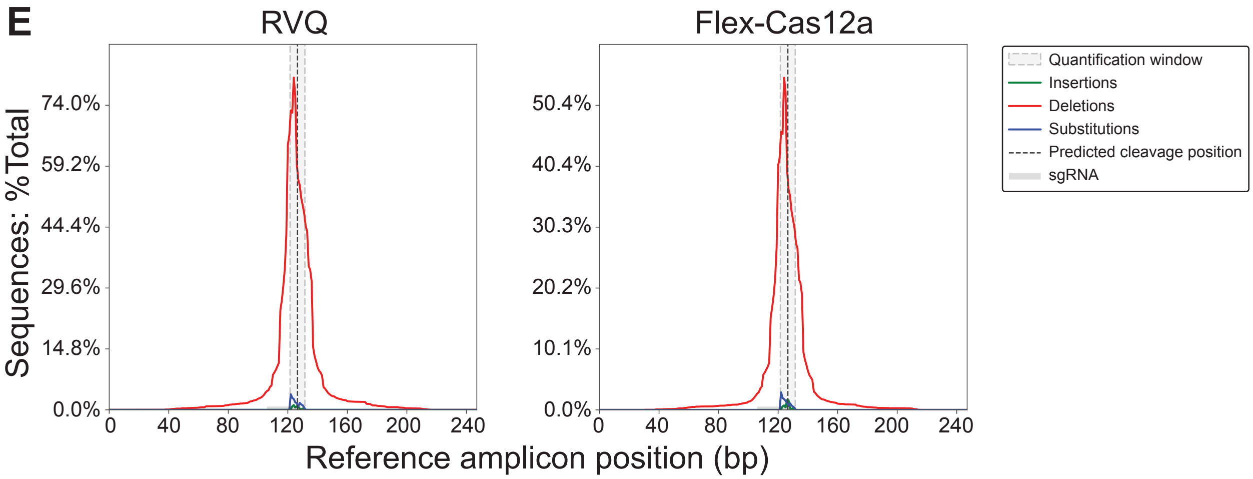

Supplementary Figure S4. Genome editing in Ai9 tdTomato NPCs and HEK293-EGFP cells mediated by direct RNP delivery or plasmid transfection.

A. Quantification of genome editing at ten target sites in tdTomato NPCs. **B.** Quantification of genome editing at ten target sites in HEK293T-EGFP cells. PAM sequence for each target is listed on top of the corresponding panel. In **A** and **B**, TTTA and TTTG are canonical PAMs, while others represent non-canonical PAMs. All the editing data were quantified using flow cytometry and are presented as mean ± SD from four independent technical replicates. neg ctrl means the cells were not treated with any proteins. Here, wt is abbreviated from wild-type, and RVQ, LbCas12a-RVQ. Panels **A** to **C** represent genome editing results via RNP nucleofection. **C and D**. Quantification of genome-editing sites in HEK293T cells transfected by 150 ng (**C**) and 200 ng plasmid (**D**). **E.** Indel profiles at the B2M target site. All sequencing reads were aligned to the reference amplicon, and indel events within the cleavage site (dotted line) were quantified at each nucleotide position. The frequency and position of deletions (red), insertions (green), and substitutions (blue) across the target region.

A

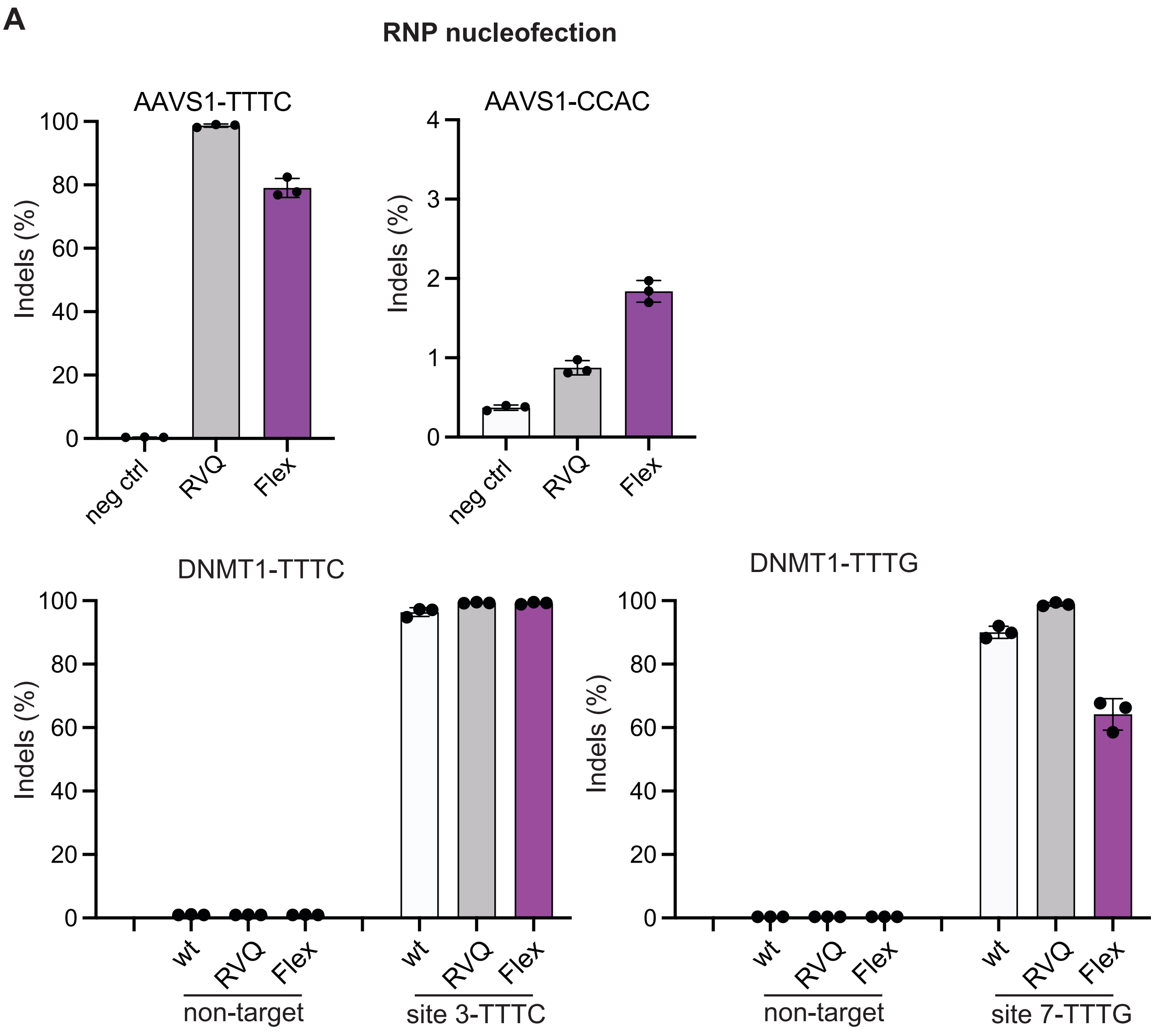

RNP nucleofection
AAVS1-TTTC
Indels (%)
100
80
60
40
20
0
neg ctrl
RVQ
Flex
AAVS1-CCAC
Indels (%)
4
3
2
1
0
neg ctrl
RVQ
Flex
DNMT1-TTTC
Indels (%)
100
80
60
40
20
0
wt
RVQ
Flex
non-target
wt
RVQ
Flex
site 3-TTTC
DNMT1-TTTG
Indels (%)
100
80
60
40
20
0
wt
RVQ
Flex
non-target
wt
RVQ
Flex
site 7-TTTG

B

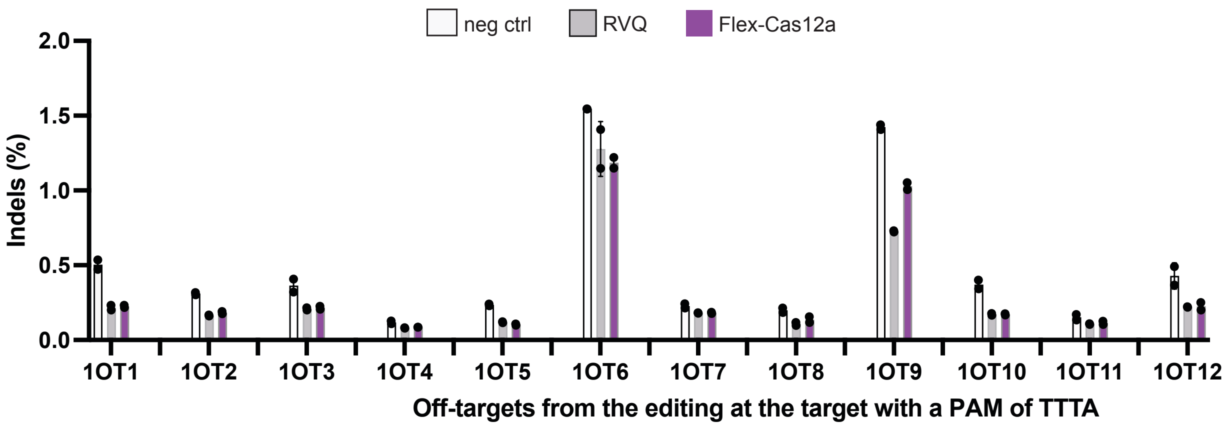
neg ctrl
RVQ
Flex-Cas12a
2.0
1.5
1.0
0.5
0.0
Indels (%)
1OT1
1OT2
1OT3
1OT4
1OT5
1OT6
1OT7
1OT8
1OT9
1OT10
1OT11
1OT12
Off-targets from the editing at the target with a PAM of TTTA

C

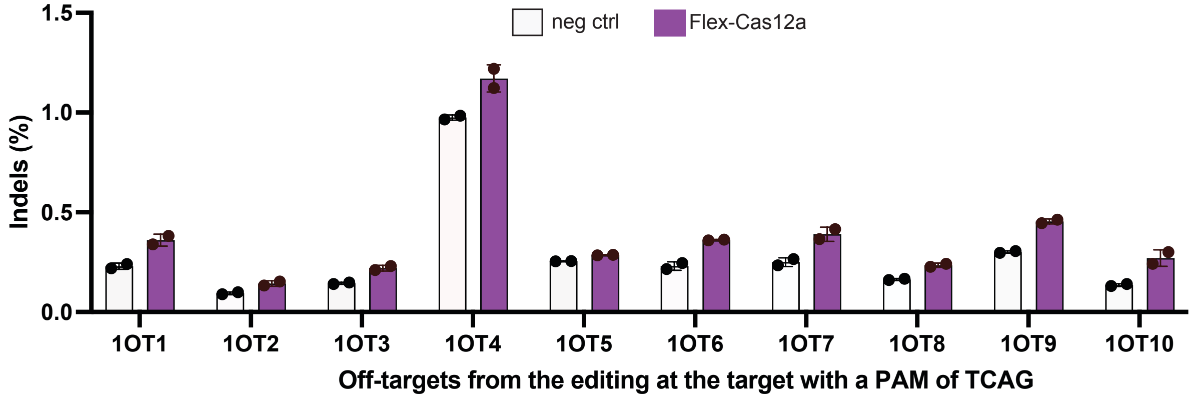
neg ctrl
Flex-Cas12a
1.5
1.0
0.5
0.0
Indels (%)
1OT1
1OT2
1OT3
1OT4
1OT5
1OT6
1OT7
1OT8
1OT9
1OT10
Off-targets from the editing at the target with a PAM of TCAG

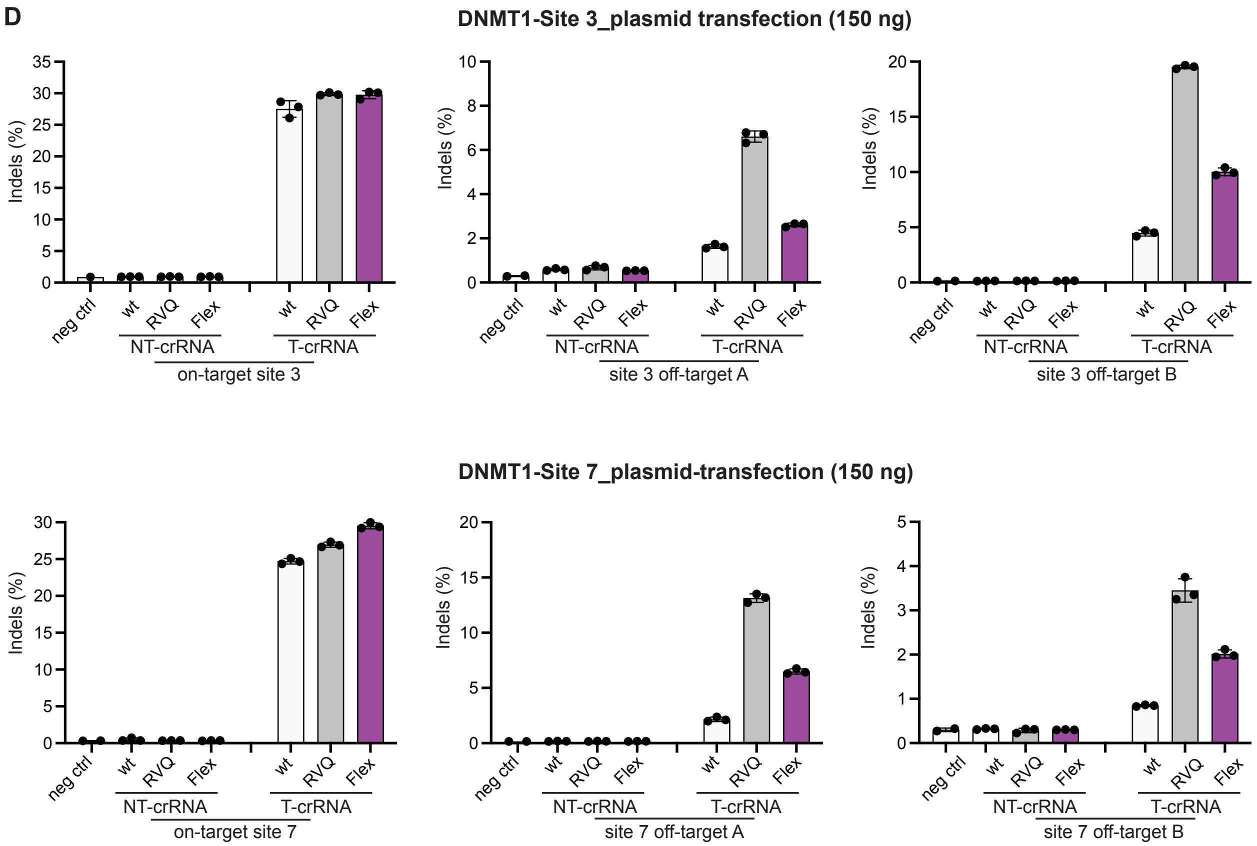

Supplementary Figure S5. Off-target analysis of Flex-Cas12a

A. Genome editing at endogenous loci mediated by RNP nucleofection. The editing levels showed by Flex-Cas12a at non-canonical PAM are significantly higher than ether wt or RVQ ($p < 0.01$). **B.** Off-target analysis of genomic DNAs isolated from the cells transfected with RNPs of LbCas12a-RVQ (RVQ) or Flex-Cas12a targeting a genomic site flanked with a 5'-TTTA-3' PAM. Twelve off-target (OT) sites were analyzed. **C.** Off-target analysis of genomic DNAs isolated from the cells transfected with RNP of Flex-Cas12a targeting a genomic site flanked with a 5'-TCAG-3' PAM. Ten off-target (OT) sites were analyzed. Each data point represents the average of two independent replicates. No off-target activity was detected in these assays. Neg ctrl means genomic DNA isolated from untreated cells. The on-target editing results for both **B** and **C** are shown in Figure 5B**. D.** Off-target analysis of LbCas12a variants. Two validated endogenous sites of DNMT1 sites, site 3 with a PAM of 5'-TTTA-3' and -site 7 with a PAM of 5'-TTTG-3', were chosen.

For each target-crRNA (T-crRNA), two validated off-target sites (off-target A and B) were analyzed. The off-target editing levels by RVQ variant are significantly higher than those of Flex-Cas12a (Flex) ($p < 0.01$) from both T-crRNAs. All on-target and off-target sequences in panels **A** and **D** are listed in Supplementary Table S2. All off-target sequences in panels **B** and **C** are listed in Supplementary Table S4. NT-crRNA = non-specific crRNA as non-targeting control.

A

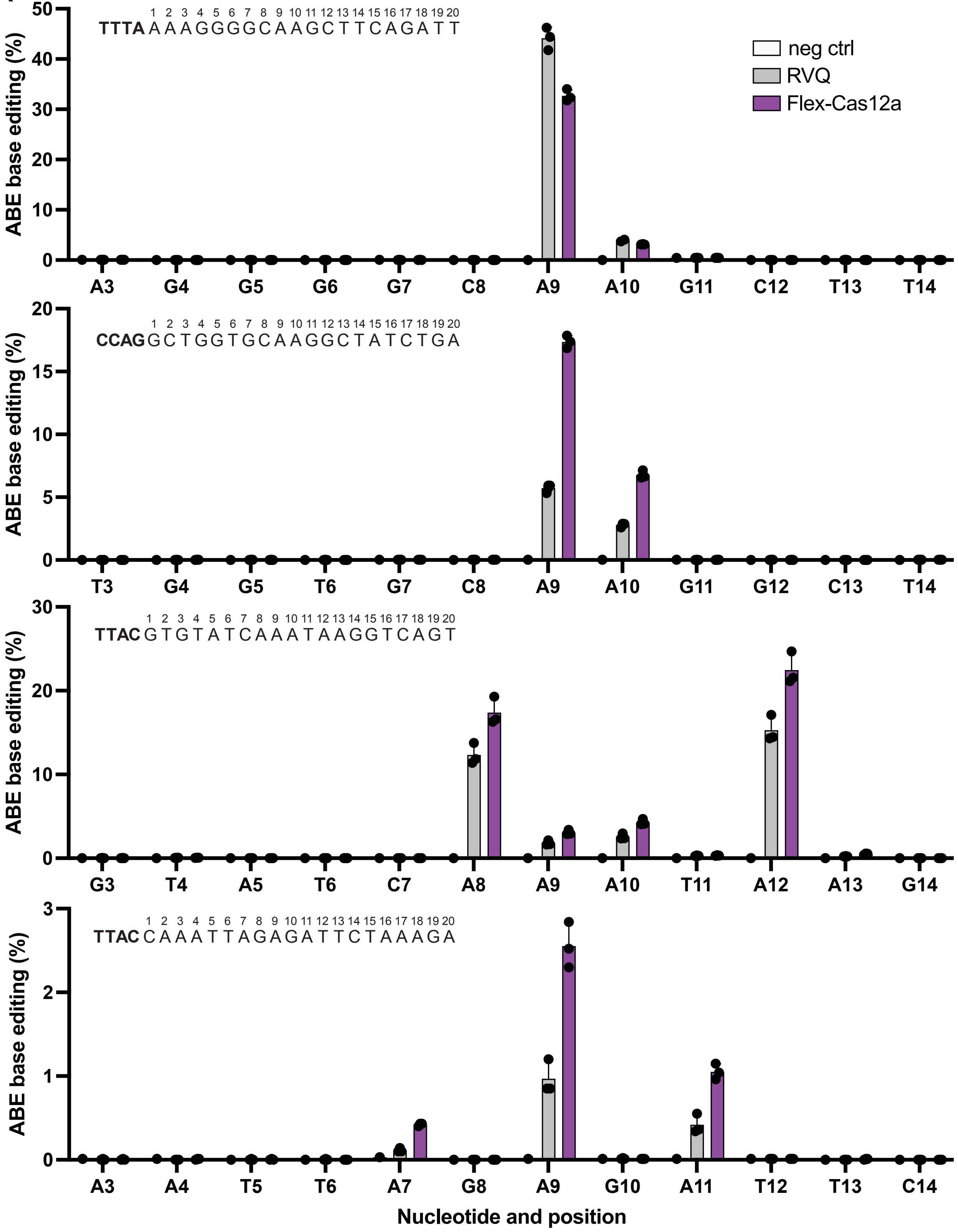

1 2 3 4 5 6 7 8 9 10 11 12 13 14 15 16 17 18 19 20
TTTA A A A G G G G C A A G C T T C A G A T T
neg ctrl
RVQ
Flex-Cas12a
ABE base editing (%)
A3 G4 G5 G6 G7 C8 A9 A10 G11 C12 T13 T14
CCAG G C T G G T G C A A G G C T A T C T G A
T3 G4 G5 T6 G7 C8 A9 A10 G11 G12 C13 T14
TTAC G T G T A T C A A A T A A G G T C A G T
G3 T4 A5 T6 C7 A8 A9 A10 T11 A12 A13 G14
TTAC C A A A T T A G A G A T T C T A A A G A
A3 A4 T5 T6 A7 G8 A9 G10 A11 T12 T13 C14
Nucleotide and position

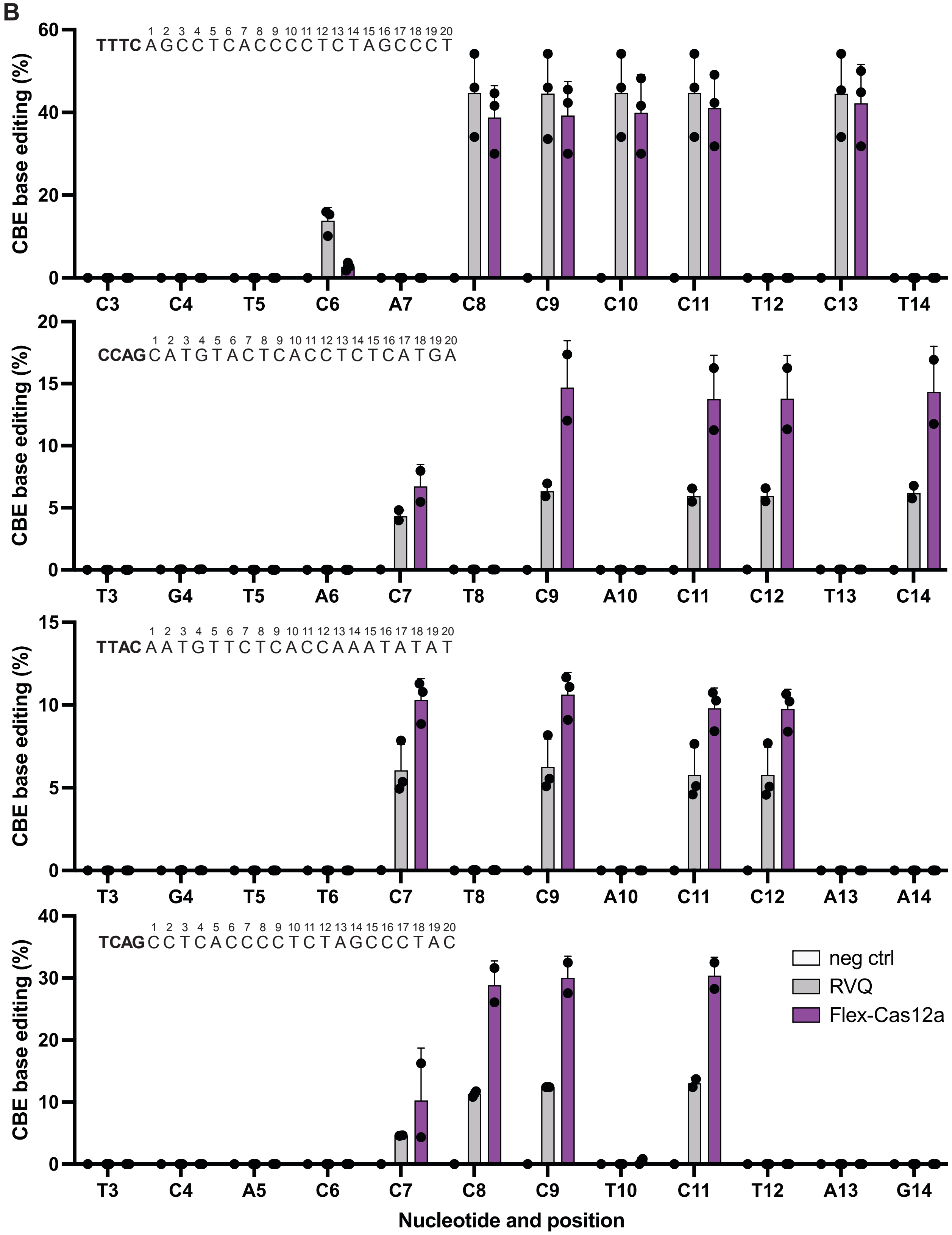
B
CBE base editing (%)
1 2 3 4 5 6 7 8 9 10 11 12 13 14 15 16 17 18 19 20
TTTC AGCCTCACCCCTCTAGCCCT
C3 C4 T5 C6 A7 C8 C9 C10 C11 T12 C13 T14
CCAG CATGTACTCACCTCTCATGA
T3 G4 T5 A6 C7 T8 C9 A10 C11 C12 T13 C14
TTAC AATGTTCTCACCAAATATAT
T3 G4 T5 T6 C7 T8 C9 A10 C11 C12 A13 A14
TCAG CCTCACCCCTCTAGCCCTAC
T3 C4 A5 C6 C7 C8 C9 T10 C11 T12 A13 G14
neg ctrl
RVQ
Flex-Cas12a
Nucleotide and position

Supplementary Figure S6. Base editing activity of Flex-Cas12a.

A. Data from ABE base editing and **B.** Data from CBE base editing. Nucleotides at each position from 3 to 14 of the target sequences are listed on the X-axis. The PAM sequence for each target is listed at the top of its corresponding panel. TTTA and TTTC are canonical PAMs, while others represent non-canonical PAMs. All the data are presented as mean ± SD from three independent replicates. RVQ is abbreviated from LbCas12a-RVQ. Target DNA sequences are listed on the top of each panel, corresponding PAMs are highlighted in bold and each position of nucleotide after PAM is also numbered.

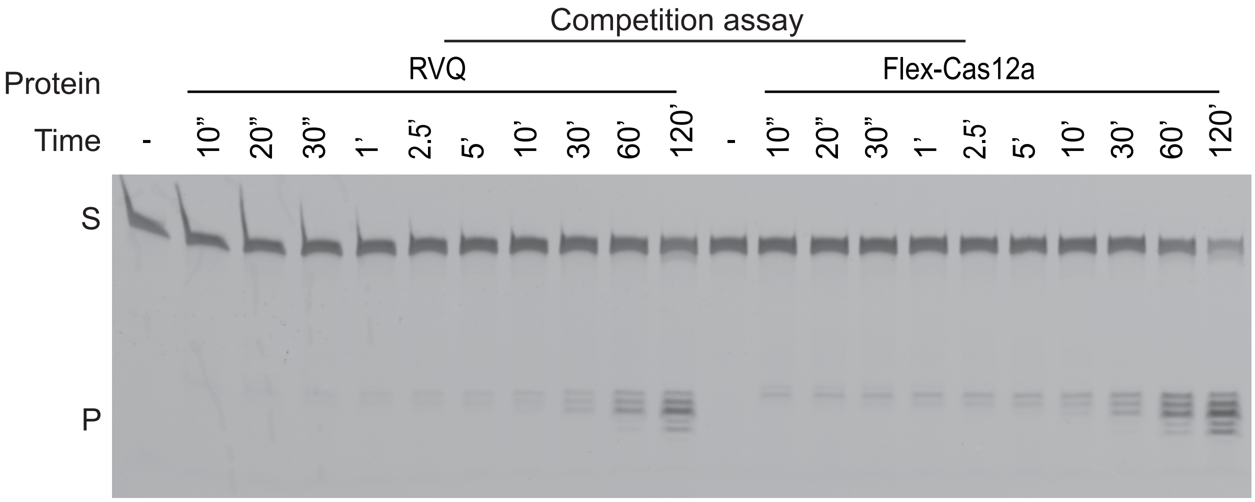

Supplementary Figure S7. Cleavage assay with a competitor DNA. In this assay, 60 nM RNP, 10 nM labeled target DNA and 360 nM competitor DNA (pUC19 plasmid) were used. Here, labeled target DNA is DNA T0 with a canonical PAM of 5'-TTTC-3' which is listed in Supplementary Table S1.

Supplementary Table S1. Sequences of DNA or RNA oligos used in this study

Supplementary Table S2. Targets of AAVs1 and DNMT1 loci and their amplicons

Supplementary Table S3. Targets and oligos used for base editing

Supplementary Table S4. Oligos and amplicons for off-targets
